# Management of Cancer Cachexia: Attempting to Develop New Pharmacological Agents for New Effective Therapeutic Options

**DOI:** 10.3389/fonc.2020.00298

**Published:** 2020-03-04

**Authors:** Gioacchino P. Marceca, Priya Londhe, Federica Calore

**Affiliations:** ^1^Department of Clinical and Experimental Medicine, University of Catania, Catania, Italy; ^2^Department of Cancer Biology and Genetics, Comprehensive Cancer Center, The Ohio State University, Columbus, OH, United States

**Keywords:** cancer cachexia, muscle tissue and adipose tissue loss, cachexia mediators, animal models, clinical trials, therapeutic strategies

## Abstract

Cancer cachexia (CC) is a multifactorial syndrome characterized by systemic inflammation, uncontrolled weight loss and dramatic metabolic alterations. This includes myofibrillar protein breakdown, increased lipolysis, insulin resistance, elevated energy expediture, and reduced food intake, hence impairing the patient's response to anti-cancer therapies and quality of life. While a decade ago the syndrome was considered incurable, over the most recent years much efforts have been put into the study of such disease, leading to the development of potential therapeutic strategies. Several important improvements have been reached in the management of CC from both the diagnostic-prognostic and the pharmacological viewpoint. However, given the heterogeneity of the disease, it is impossible to rely only on single variables to properly treat patients presenting this metabolic syndrome. Moreover, the cachexia symptoms are strictly dependent on the type of tumor, stage and the specific patient's response to cancer therapy. Thus, the attempt to translate experimentally effective therapies into the clinical practice results in a great challenge. For this reason, it is of crucial importance to further improve our understanding on the interplay of molecular mechanisms implicated in the onset and progression of CC, giving the opportunity to develop new effective, safe pharmacological treatments. In this review we outline the recent knowledge regarding cachexia mediators and pathways involved in skeletal muscle (SM) and adipose tissue (AT) loss, mainly from the experimental cachexia standpoint, then retracing the unimodal treatment options that have been developed to the present day.

## Introduction

Cancer-associated cachexia (CC) is a paraneoplastic syndrome whose outbreak is governed and driven by inflammation. It involves various organs and is characterized by changes in body composition (1–3). Although loss of appetite is frequently associated with cancer cachexia, tissue wasting under cachectic conditions occurs through different modalities compared to those triggered by starvation, which primarily affects the adipose tissue (AT). The major feature of cachexia is, instead, represented by loss of skeletal muscle (SM) mass, not necessarily accompanied by AT wasting (4).

Loss of muscle mass in cachectic individuals is due to altered proteostasis, which results from a combination of enhanced proteolysis and decreased protein synthesis (5, 6). Here, the high rate of proteolysis largely depends on upregulation of ubiquitin–proteasome and autophagy–lysosomes pathways, while calpains and caspases have been found to contribute to a lesser extent, though valuably. For instance, the calpains-mediated protein degradation is thought to act upstream of the ubiquitin–proteasome pathway, thus allowing the release of myofilaments from the myofibrils and their subsequent ubiquitylation and degradation (7).

Patients with CC often manifest generalized hypermetabolism with lower energy intake and higher energy expenditure. In particular, anomalous increases in resting energy expenditure (REE) are nowadays considered the major contributor to energy consumption. In this context, systemic inflammation and changes in the immune system both represent important determinants of this condition, albeit the precise mechanisms remained elusive (8, 9). Increases in energy expenditure are also explained by enhanced thermogenesis and “browning” of white adipose tissue (WAT), which consists of a rapid increase of beige adipocytes (10, 11). Moreover, WAT of cachectic individuals can undergo to consistent dissolution of fatty acids and glycerol. Such an event seems to occur in consequence to increased lipolytic activity of adipose triglyceride lipase (ATGL) and hormone-sensitive lipase (HSL), two key enzymes involved in catabolism of triglycerides. On the contrary, some studies suggest that no significant downregulation in lipogenesis or expression/activity of lipoprotein lipase (LPL) occur in patients with CC (12, 13).

Incidence and prevalence of CC are not homogenous across cancer patients, but they rather vary depending on the tumor type and stage. CC has been mainly associated with incurable cancers and is highly prevalent at the end of life (14, 15); however, it can also occur in curable cancers and may be reversed by properly treating the underlying tumor (16). Meanwhile, this syndrome is notoriously influenced by additional endogenous and environmental factors, such as comorbidities, genetic risk factors, sex, age, and anti-cancer treatment (17–19). A report based on information provided by two independent studies showed a prevalence of ~70% for pancreatic cancer, ~60% for gastro-esophageal and head-neck cancers, and 50–40% for non-small-cell lung cancer, colorectal cancer, and certain hematological malignancies (20). Similarly, a recent systematic review (15) reported very high risk (80–90%) of developing cachexia for patients with liver, pancreatic and lung cancers, followed by head-neck, gastric and colorectal cancers (60–70%). On the contrary, patients with thyroid, breast and prostate cancers and melanoma of the skin represented the groups at lowest risk (~20%). In the same study, cachexia was defined for the first time an orphan disease (15).

Approximately 20–30% of mortalities in cachectic tumor patients are thought to derive from cachexia rather than from the tumor burden itself (6, 21). In addition, cachexia can lead to lower responsiveness to anticancer therapies, worsening of life quality and poor prognosis in advanced tumor patients (14, 22, 23). In this regard, it has been reported that treating cachectic patients with conventional chemotherapeutic regimens further enhances hypercatabolism of SMs and could cause changes in fat and bone mass. This would exacerbate the pathological condition, thus requiring dosage limitation or therapy interruption (3).

Clinically, combinations of metastatic tumor, muscle wasting, debilitation, and refractoriness to chemotherapy seriously limit benefits from treatments of cachexia, even when multimodal options are adopted. Thus, the identification of reliable diagnostic markers, predictive of CC, is of fundamental importance to prevent the patient's physiological decline. According to the most recent consensus report, published by Fearon et al., the current standard diagnostic criterion for cachexia is represented by a weight loss >5% over the past 6 months, or any degree of weight loss >2% in individuals showing a Body Mass Index (BMI) <20 or sarcopenia (14). In the same report, a classification criterion was proposed to clinically subdivide CC into three stages, aiming to properly treating cachectic tumor patients. In line with the diagnostic criterion, such classification would be based on three established parameters of cachexia, i.e., percentage of weight loss, BMI values and metabolic changes (14). Specifically, patients manifesting anorexia, impaired glucose tolerance and weight loss ≤ 5% over the past 6 months would be classified as pre-cachectic. Patients with systemic inflammation, BMI < 20, weight loss > 5% over the past 6 months and ongoing weight loss of more than 2% would be classified as cachectic. Absence of responsiveness to anticancer treatments or preterminal cancer stage would instead determine refractory cachexia (14). However, this classification cannot currently be given as officially accepted by clinicians, but is rather to be considered as a proposal under evaluation. Indeed, additional diagnostic parameters such as hemoglobin (Hb) and albumin levels were previously proposed (4). The same parameters were then re-evaluated by two more recent studies, with the addition of C reactive protein levels (24, 25). However, none of these were included in the diagnostic definition of CC neither in its classification by the last consensus, although it was stated that several other components of cachexia (thus including the aforementioned ones) should be further evaluated (14).

Interestingly, some research groups investigated on possible causative genetic variants underlying CC or cachexia-related appetite loss in cancer patients [e.g., (26, 27)]. Although these studies put into evidence dozens of single nucleotide polymorphisms as potentially involved in such processes, no significant findings were brought out. Moreover, some discrepancies emerged when results from some of these studies were compared to one another (26). Nonetheless, a role for genetics in the pathophysiology of CC cannot be excluded, since it should be considered that this type of analysis is in its early stage. Thus, further genome-wide approaches are needed, considering the complexity and the variability of the syndrome.

From the molecular standpoint, numerous *in vitro* and *in vivo* studies have demonstrated that several pro-inflammatory cytokines, toll-like receptors (TLRs) and growth/differentiation factors (GDFs) act as mediators of CC. In general, most of these molecules are purposely used as signaling molecules in cell-to-cell communication and mechanisms involved in innate immunity, and exert pleiotropic effects. For instance, cytokines are primarily produced by immune cells, although several other cells of the organism as well as tumor cells are capable to express them (28). In the pathogenesis of cancer, the tumor-induced inflammatory response leads to expression and secretion of a number of immune-suppressive and pro-inflammatory cytokines by immune cells, aiming to eradicate tumor cells from the host (29). However, inappropriate accumulation/regulation of leukocytes in the tumor site can cause an imbalance between pro- and anti-inflammatory mechanisms, eventually leading to chronic inflammation and subsequent immunosuppression (30), as occurs in advanced cancer patients. As a result, the chronic presence of such mediators of inflammation in both the tumor microenvironment and circulation causes systemic deregulations and metabolic dysfunctions in the host, including CC (2, 29).

## Mediators of CC: What Have We Learned From *in vitro* and *in vivo* Studies

Experimental research on CC has experienced an exponential increase in terms of gained knowledge during the last three decades. In particular, the identification of several endogenous factors functioning as mediators of CC and the uncovering of their relative mechanisms of action has led to the achievement of important frontiers in this field of oncology. This has allowed the development of potential effective pharmacological agents for the clinical management of this metabolic syndrome (31). Intriguingly, we now know that several of these effectors share the same or similar metabolic effects, and that most often they exhibit synergic effects when administered together. Moreover, most of them are simultaneously involved in both SM and AT depletion, though exerting a distinct role depending on the target tissue (see next section).

### Tumor Necrosis Factors

Tumor necrosis factor alpha (TNFα, also known as cachectin) has long been shown to play a role in murine models of CC (32, 33). Albeit normally involved in acute phase reaction triggering and apoptosis, TNFα can also promote tumorigenesis and metastasis, and has been shown to act as an autocrine growth factor for various tumor types (34). Early studies showed that TNFα had the ability to inhibit differentiation of both skeletal myocytes and adipocytes (35, 36), while it caused reduced protein content and higher degradation of myofibrillar proteins in differentiated skeletal myocytes, in a time- and dose-dependent manner (37, 38). However, later experiments demonstrated that TNFα alone was not sufficient to cause a significant dysfunction of skeletal myofibers in differentiated myocytes, but a synergic action with other cytokines, such as interferon gamma (INFγ), was required to produce valuable effects [e.g., (35, 39)]. More recent studies have reported similar results for a structural homolog of TNFα, i.e., TNF-related weak inducer of apoptosis (TWEAK, also known as TNFSF12), which presents overlapping signaling functions with the former (40, 41).

### Interleukins

Some of the cytokines belonging to the class of interleukins (ILs) have been shown to significantly contribute to tumor growth and CC. First and foremost, circulating interleukin-6 (IL-6) is recognized as one of the main factors leading to the outbreak of cachexia. For instance, significant concentrations of IL-6 were detected in the serum of cachectic mice transplanted with a cachexia-inducing colon-26 adenocarcinoma (C26) subtype, where serum level of IL-6—but not that of TNFα–correlated with severity of the pathological status (42). Yet, high constitutive levels of circulating IL-6 caused suppression of muscle protein synthesis at the initial stage of cachexia in a different murine model (43). On the contrary, attenuation of the IL-6 signaling was shown to abolish key features of CC (42, 44), although such inhibition was not sufficient to reverse the process (43). In a similar manner, it has been evidenced a role for IL-6 in induction of energy expenditure and loss of fat mass by promotion of WAT browning. In fact, the knock-out of IL-6R in cachectic mice transplanted with B16 melanoma cells showed partial though significant reduction of WAT browning when compared with control mice (11). At the same extent, the genetic blockade of IL-6 was found to be critical in a syngeneic graft model with C26 cancer cells, as it prevented WAT browning and cachexia (11).

Leukemia inhibitory factor (LIF) is another IL-6 family member, involved in a variety of distinct biological processes including inflammation, cell growth, differentiation, neural development, and hematopoiesis (45, 46). As in the case of IL-6, LIF was previously reported to induce muscle wasting in various animal models (47, 48). A recent study further demonstrated the involvement of tumor-secreted LIF in myotube atrophy in a C26 cancer-induced cachexia model (49). Here, elevated circulating levels of this interleukin induced expression of atrophy-related genes. On the contrary, immunological blockage of circulating LIF prevented the triggering of such phenomenon (49). LIF has recently been shown to be implicated in cachexia-associated lipolysis as well (50), whereby interaction between this interleukin and its cognate membrane receptor LIFR-α caused enhanced expression of genes involved in lipids catabolism. Interestingly, the same study revealed a double mechanism of action for LIF, since it sustained fat mass loss through an equal combination of peripheral (i.e., directly exerted on adipocytes) and central (i.e., exerted on the hypothalamus) contributions. In particular, the latter mechanism of action was counterbalanced by leptin signaling (50).

Similarly, to IL-6, IL-1 alpha (IL-1α) was initially evidenced as a factor capable to induce protein breakdown in isolated SM during systemic inflammation (51). Administration of IL-1α was found to induce cachexia together with anorexia by causing accelerated SM protein wasting in a rat model (52). Accordingly, the pharmacological blockade of IL-1α receptor reduced tumor growth and slowed down the development of CC in methylcholanthrene-induced sarcoma (MCG 101)-bearing mice (53).

A more recent study demonstrated how IL-1 beta (IL-1β) increased SM catabolism in a rodent model of CC by promoting a cachexia-associated gene expression pattern in the hypothalamic–pituitary–adrenal (HPA) axis, differently from IL-6 and IL-1α (54). Few other studies on experimental cachexia have raised the possibility for other interleukins to be involved in the onset of cachexia, as is the case of IL-10 and homodimeric IL-12 (55), although with a lesser extent of evidence.

### Interferon Type II

INFγ, the only member of the type II class of interferons, has a critical role in innate and adaptive immunity against viral infections, promotes activation of macrophages and exerts mild antiproliferative effects on certain cell types (56). Nonetheless, several studies have reported a role for this cytokine in enhancement of tumor growth, metastasis (57, 58) and development of CC (59). Nude mice injected with genetically engineered ovary tumor cells (CHO) producing murine IFN-γ developed severe cachexia, contrary to those inoculated with the parental tumor cell line (60). Accordingly, when mice injected with CHO/INFγ cells were treated with anti-INFγ monoclonal antibodies, development of cachexia was prevented (60). In line with this, early or late treatment of Lewis lung carcinoma (LLC)-bearing mice with anti-INFγ monoclonal antibodies was shown to counteract progression of cachexia (61). Importantly, both of these studies reported that here AT, but not SM, was the main target of INFγ (60, 61).

### MicroRNAs

Over the last decade, several proofs were gained about the involvement of non-coding RNAs, in particular microRNAs (miRNAs), in loss of lean and fat mass under cachectic conditions. In their mature form, miRNAs are ~21–23 nucleotides in length and exert a well-recognized role in gene regulation. In particular, miRNAs have been extensively studied as biomarkers for histological classification, disease prognosis, clinical response to treatments and diagnosis of cancer (62, 63). Besides exerting their action within the cell, miRNAs can be released into extracellular fluids and are referred to as extracellular or circulating miRNAs. At the same extent of intracellular miRNAs, circulating miRNAs are capable to modulate gene expression in recipient cells, determining, adjusting or deregulating cells' physiological status (64). Moreover, secreted miRNAs can have a role in cell-to-cell communication [e.g., (65, 66)]. As CC mediators, miRNAs seem to exert their action by favoring cell apoptosis, protein degradation, or causing downregulation of anabolic processes. For instance, circulating miR-203a-3p secreted by metastatic human colorectal cancer contributed to exacerbation of myopenia in pre-operative cancer patients by targeting survivin (BIRC5), a negative regulator of caspase-dependent apoptosis (67). MiR-21-5p and−206, instead, played an important role in the onset of muscle atrophy in mice under distinct muscle-debilitating conditions, including CC. This was due to the miRNA-mediated inhibition of the translational initiator factor eIF4E3 and transcription factor Yin Yang 1 (YY1), involved in mitochondrial biogenesis (68). Yet, mir-155-5p secreted by human breast cancer cells was proven to promote WAT browning and increased BAT thermogenesis but not lean mass loss. This was due to the targeting of peroxisome proliferator-activated receptor gamma (PPARγ), a well-known regulator of glucose metabolism and fatty acid storage (69).

### Toll-Like Receptors

Toll-like receptors (TLRs) are other essential components of the innate immune response. TLRs are present either on the cell surface or in endosomes, and are capable to interact with microbial components, danger-associated self molecules or non-self nucleic acids presenting well-defined patterns. Once stimulated by such interactions, TLRs activate two possible signaling pathway leading to downstream immunogenic gene expression (70). One recent study has evidenced differential expression of TLR genes in cachectic LLC-bearing mice compared to non cachechtic controls (71). Moreover, results from the same study suggested that increased expression of TLRs mRNA may depend on factors secreted by tumor cells, and that different tumors, such as LLC and C26, are likely to induce distinct pattern of TLR expression.

Among TLRs, TLR4 belongs to the cell-surface group and is well-known to possess high affinity for lipopolysaccharide (70). TLR4 was initially shown to act as a master regulator of inflammatory muscle catabolism, as its exposition to lipopolysaccharide caused significant loss of myofibrillar proteins and mitochondria in both cultured and *in vivo* murine muscle cells (72). Later, this receptor was demonstrated to be directly implicated in myotube atrophy in LLC-bearing mice, causing cachexia. In this murine model, activation of TLR4 was caused by cellular uptake of high levels of heat shock protein 70 (Hsp70) and Hsp90, secreted by tumor cells through extracellular vesicles (EVs) (73). In turn, activation of TLR4 correlated with evident muscle wasting and increased levels of circulating TNFα and IL-6 (73). TLR4 was also reported to be involved in remodeling of AT in LLC-bearing mice. In fact, it was demonstrated that either genetic ablation or pharmacological inhibition of TLR4 in cachectic mice caused suppression of adipocytes atrophy and also reduced macrophage infiltration into the AT (74).

TLR7/8 is mainly located at the endosomal level and recognizes viral single-stranded RNA molecules (70). TLR7/8 was found to have a significant impact in experimental CC after being activated by EV-derived cargo. Specifically, our group demonstrated that murine TLR7 was capable to interact with EV-derived miR-21-5p secreted by lung and pancreatic cancer cells. Once activated, TLR7 induced muscle loss by promoting apoptosis in cultured myocytes in a JNK-dependent manner. On the contrary, myocytes from TLR7^−/−^ LLC-bearing mice showed significantly reduced cell death (66). Recent experiments confirmed these results (75) and demonstrated that also tumor-secreted miR-29a-3p was capable to interact with murine TLR7, inducing cell death even with higher effectiveness than miR-21-5p (75).

A role in development of cancer cachexia has been recently suggested for TLR5 as well (76). In fact, activation of this receptor, which is expressed on the plasmatic membrane of certain gastric cancer cells, seemed to contribute to the onset of CC, possibly due to TLR5-mediated overexpression of LIF (76).

### Cytokines of the Transforming Growth Factor β Superfamily

Besides pro-inflammatory cytokines and TLRs, regulatory proteins belonging to the transforming growth factor β (TGFβ) superfamily of cytokines have been identified as potential actors exacerbating SM atrophy in cachexia. Among these, Myostatin (Mstn) and Activin A (ActA) present a partial overlap in their signaling function, since both are capable to interact with either of the Activin type II receptors (ActRIIA or ActRIIB), expressed on the surface membrane of skeletal myocytes (77). Mstn (also known as GDF8) is primary synthesized by SM cells and functions as an autocrine factor causing a strong downregulation of myogenesis (78, 79), while inhibition of its expression/function leads to a dramatic increase in muscle mass (80, 81). Following this line, later studies demonstrated that Mstn mRNA expression levels increased up to ~50% in experimental models of CC, while expression levels of MyoD, a myogenic transcription factor indispensable for myoblast fusion and SM development, decreased of ~45% in comparison with controls (82, 83). In contrast, when synthetized antisense RNA oligonucleotides were used to target the Mstn mRNA *in vivo*, expression levels of both Mstn and MyoD tended to revert toward the normal phenotype, and led to a significant increase in muscle mass (83).

Differently from Mstn, ActA is expressed by a variety of cell types, has a wide range of regulatory functions and is negatively modulated by several factors including Inhibin (Inhb) and Follistatin (FS) (84). In the context of muscle wasting, concentrations of circulating ActA seem to be particularly increased during acute inflammation or in certain metastatic cancers (85, 86). Since ActA can reproduce the biological action of Mstn by binding to the ActRII receptors, knockdown experiments for Inhb caused dramatic loss of both lean and fat mass in mice, leading to death (87). Similarly, increase of local or circulating concentrations of ActA induced by direct methods caused muscle atrophy in treated mice (88). On the contrary, the pharmacological blockade of ActRIIB or administration of its soluble decoy forms (sActRIIB) in multiple rodent models of CC reversed atrophy of skeletal and cardiac muscles and prolonged survival of treated mice, albeit it had no effects on tumor growth or loss of AT (87, 89).

### Parathyroid Hormone-Related Peptide

Two recent works highlighted the role of parathyroid hormone-related peptide (PTHrP) in WAT browning under cachectic conditions (10, 90). Besides being expressed in kidney and bone (91), PTHrP can be overexpressed by many tumor types and acts as an endocrine effector capable to induce thermogenic gene expression in adipocytes. Moreover, its presence in the circulation correlates with a greater degree of wasting in individuals with metastatic cancer (10). PTHrP induced thermogenesis and hypermetabolism of the AT in a murine model of LLC. The evidence was given by the fact that treatment with PTHrP antibodies strongly prevented the tumor-induced AT browning in comparison to the control group (10). In addition, treatment with PTHrP antibodies also impaired LLC tumor-induced muscle wasting, atrophy of muscle fibers, and atrophy-related gene expression in treated mice (10). In a later study, the same research group corroborated the previous results demonstrating a key role for the PTHrP receptor (PTHR) in triggering browning and thermogenesis following the binding to its ligand (90). Mice knockout for PTHR in their fat tissue were resistant to cachexia driven by the LLC tumor.

### Adipokines

Zinc-α2-glycoprotein (ZAG) is an adipokine functioning as a lipid mobilizing factor (LMF). ZAG is usually expressed by differentiated adipocytes (92), albeit it was reported that other tissues express it as well (93), including several cancer cells (94). ZAG expression has been reported to be markedly elevated in AT of mice transplanted with cachexia-inducing tumor (93), whereby this phenomenon has been positively correlated with increased lipolysis and subsequent fat loss (95, 96). As a counterevidence, knockout of ZAG caused significant increases in bodyweight of mice fed with standard or lipid-rich diet when compared with wild-type controls. Similarly, the same group of ZAG-deficient mice showed significant decrease in adipocyte lipolysis in response to treatment with agents that increase cAMP (97). One study carried out on cachectic patients with gastrointestinal cancer showed that ZAG is primarily produced and secreted by WAT, and correlates with nutritional status in both malignant and nonmalignant conditions. However, it seems that neither WAT nor tumor cells secretory activity lead to significant increases of circulating levels of this adipokine. Thus, ZAG should be considered as a lipolysis-promoting factor locally produced (94).

## Putting Pieces Together: Pathways Underlying CC

### Dysregulated Pathways in SM

From a systematic standpoint, all the aberrant metabolic features characterizing CC are caused by activation of few distinct signaling pathways, triggered by the interaction between the aforementioned mediators and their cognate receptors.

Under normal conditions, myofibrils, the main structural components of myocytes, physiologically undergo to a balanced protein turnover. Under cachectic conditions, instead, excessive rates of myofibril breakdown and low rates of protein synthesis are typically observed in patients' SM, resulting in muscle weakness, fatigue, reduced tolerance to chemotherapy and low quality of life (QoL) (1, 3). Here, the ubiquitin-proteasome pathway, which takes part in breakdown of short-lived and regulatory proteins, is known to play a relevant role (98). As its name suggests, this proteolytic pathway is essentially dependent on the presence of active enzymes involved in protein ubiquitylation, which include the well-known family of E3 ubiquitin ligases. Among these, muscle RING finger-containing protein 1 (MURF1, also known as TRIM63) and muscle atrophy F box protein (MAFbx, also known as Atrogin-1) are defined as muscle-specific E3 ubiquitin ligases and are the most widely studied in the case of cachexia-associated muscle loss. Both these enzymes mediate myofibrillar protein breakdown by acting on several components of the sarcomeric thick filament, including myosin heavy chain (MHC), and are thought to interfere with processes related to protein synthesis (99). The proinflammatory cytokines TNFα, TWEAK and IL-1 have been shown to cause increased expression of both these E3 ubiquitin ligases via the nuclear transcription factor kappa B (NF-kB) signaling and the p38/CCAAT/enhancer-binding protein β (p38/C/EBPβ) pathway, respectively (35, 41, 100). Activation of the NF-kB signaling was reported to cause overexpression of paired box 7 (PAX7) in myocytes (101). The latter is a transcription factor playing an essential regulatory role in myogenesis, being responsible for repression of MyoD and myogenin transcriptional activity. In line with this, it has been demonstrated that NF-κB–dependent upregulation of PAX7 impairs the regenerative ability of myogenic cells and drives toward muscle wasting under tumor conditions (101).

A different signaling pathway is instead activated by IL-6. In particular, this interleukin signals via its membrane receptor IL-6R, which in turn forms a heterodimer with its transducing subunit (gp130). This leads to activation of the Janus kinase/signal transducer and activator of transcription (JAK/STAT) pathway, with subsequent translocation of activated STAT3 into the nucleus. This event contributes to the regulation of expression of the E3 ubiquitin ligases and autophagy genes (102). STAT3 also induces expression of C/EBPδ, a transcription factor that promotes expression of Mstn under cachectic conditions (102). Indeed, a recent work revealed that, at the same extent of IL-6, TNFα, and INFγ synergistically activate STAT3 by promoting its JAK-mediated phosphorylation independently from IL-6 (103). This demonstrates that NF-kB and STAT3 both respond to the same upstream signaling, and thus cooperate amplifying the signal and promoting expression of pro-cachectic genes.

Although the precise mechanism have remained elusive, it is known that proinflammatory cytokines also suppress activity of the RAC serine/threonine-protein kinase (AKT), which is downstream of insulin signaling and central to many cellular processes. AKT negatively modulates the transcriptional activity of forkhead box protein O1 (FoxO1) and FoxO3, which control the expression of genes involved in metabolic homeostasis, including that of MURF1 and MAFbx (104, 105). In particular, under physiological conditions, AKT phosphorylates the FoxOs proteins, preventing their nuclear translocation. Moreover, AKT inhibits activity of tuberous sclerosis complex 2 (TSC2), a main inhibitor of the mammalian target of rapamycin complex 1 (mTORC1), which is notoriously involved in functions like protein synthesis, pentose anabolism and blockage of autophagy (106). To the contrary, cytokine-mediated suppression of AKT causes downregulation of mTORC1 activity and favors FoxOs dephosphorylation and nuclear location, thus promoting miofibrillar degradation (107, 108). Interestingly, it was recently reported that FoxOs also induce expression of a third muscle-specific E3 ubiquitin ligase, termed specific of muscle atrophy and regulated by transcription (SMART) (108).

One further negative regulator of FoxO3 is the peroxisome proliferator-activated receptor gamma coactivator 1-alpha (PGC1α), a master regulator of mitochondrial biogenesis and transcriptional coactivator regulating expression of genes involved in energy metabolism (109). PGC1α was demonstrated to be downregulated in muscles of cachectic tumor-bearing mice, while its transgenic expression allowed muscle recovery *in vivo* (109).

Aberrant activation of signaling pathways downstream of TLRs is also known to contribute to cancer progression and overproduction of proinflammatory cytokines underlying cachexia (110). Some studies demonstrated that the notorious TLR/myeloid differentiation factor 88 (TLR/MyD88) signaling (70) mediates skeletal muscle wasting during CC (71, 111). Previous studies suggested that the mechanism involved in such phenomenon is the TLR/MyD88-mediated activation of NF-kB signaling (112–114). Indeed, *in vivo* experiments carried in a recent work partially confirmed such hypothesis by demonstrating that ablation of MyD88 in LLC-bearing mice inhibited tumor-induced activation of NF-kB in SMs (71). However, the authors showed that most of muscle mass loss in this murine model was due to activation of the TLR4/MyD88/inositol-requiring protein 1α (IRE1α)/X-box-binding protein 1 (XBP1) axis. Both IRE1α and XBP1 are linked to the unfolded protein response signaling, which is activated following accumulation of misfolded proteins or dysregulations in calcium levels within the SM. In particular, the spliced form of the transcription factor XBP1 was demonstrated to be a potent promotor of expression of proinflammatory cytokines and autophagy-related genes, while its ablation resulted in significant attenuation of loss of lean mass both *in vitro* and *in vivo*. Importantly, the authors showed that this mechanism is likely to involve not only TLR4, but also some other TLRs, including TLR7 (71).

Through their binding to ActRII receptors, TGFβ-family members like Mstn and ActA promote protein degradation via p38/JAK-mediated phosphorylations and activation of small mother against decapentaplegic (SMAD) signaling (115). In particular, it is known that, in the case of myoblasts and myocytes, Mstn specifically interacts with the ActRIIB receptor, subsequently inducing the assembly of type-I receptor ALK4. This in turn leads to activation of the SMAD2/3/4 complex (115, 116). Once assembled, the SMAD complex is translocated to the nucleus and acts as a transcription factor favoring expression of genes related to protein degradation and inhibiting that of genes involved in protein synthesis and proliferation (115).

**Figure 1:**
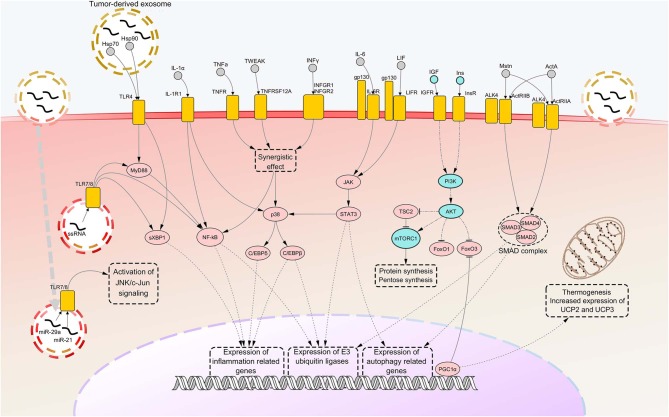
Notorious molecular mechanisms underlying skeletal muscle wasting during cancer cachexia. Atrophy of skeletal muscle in cancer cachexia is due to aberrant activation of specific signaling pathways, consequent to the binding of factors secreted by the tumor, the stroma or the immune system to their cognate receptors. Most of such signaling pathways converge toward activation of selective transcription factors, causing their nuclear translocation and binding to promoters of cachexigenic genes. These include genes encoding cytokines [e.g., tumor necrosis factor alpha (TNFα) and interleukin 6 (IL-6)], inflammation-related receptors [e.g., toll-like receptor 4 (TLR4) and TLR7], myokines [e.g., myostatin (Mstn)], muscle-specific E3 ubiquitin ligases and autophagy-related proteins. Such events potentiate inflammatory processes at the local level and cause the breakdown of myofibrillar proteins, impairing the contractile function of skeletal muscles. Several cachexia-inducing factors are known to exert their cachectigenic effect by acting synergistically, as in the case of TNFα and interferon gamma (INFγ). To the contrary, downregulation occurring in the insulin (Ins) and insulin growth-like factor (IGF) signaling determines a decrease in mTOR-dependent protein synthesis, due to upstream downregulation of RAC serine/threonine-protein kinase (AKT) activity. In normal conditions, AKT functions also as a negative regulator of forkhead box protein O1 (FoxO1) and FoxO3 transcription factors, preventing their nuclear translocation. Thus, consequent to AKT downregulation under cachectic conditions, both these FoxOs localize into the myonucleus and induce transcription of autophagy components and muscle-specific E3 ubiquitin ligases. Peroxisome-proliferator-activated receptor-gamma co-activator 1-alpha (PGC1α), overexpressed during cancer cachexia, is known to inhibit FoxO3 binding to the DNA and cause enhance expression of genes involved in energy metabolism and mitochondrial biogenesis. Upregulated nodes are colored in pink and connected with other nodes through continuous edges. Downregulated nodes are colored in azure and connected with other nodes through dash-dot edges. Dashed edges represent connections between transcriptional/co-transcriptional factors and gene expression. ActA, activin A; ActIIR, activin type 2 receptor; ALK4, activin receptor type-1B; C/EBP, CCAAT/enhancer binding protein; c-Jun, proto-oncogene c-Jun; Gp130, glycoprotein 130; Hsp, heat shock protein; IL-1a, interleukin 1 alpha; IL-1R1, interleukin 1 receptor 1; IL-6R, IL-6 receptor; INFGR, INFγ receptor; JAK, Janus kinase; JNK, c-Jun N-terminal kinase; LIF, leukemia inhibitory factor; LIFR, LIF receptor; miR, microRNA; mTORC1, mammalian target of rapamycin complex 1; MyD88, myeloid differentiation factor 88; NF-kB, nuclear transcription factor kappa B; p38, p38 mitogen-activated protein kinase; PI3K, Phosphoinositide 3-kinase; SMAD, small mother against decapentaplegic; STAT3, signal transducer and activator of transcription 3; sXBP1, spliced isoform X-box-binding protein 1; TNFR, tumor necrosis factor receptor; TNFRSF12A, TNF Receptor Superfamily Member 12A; TSC2, tuberous sclerosis complex 2; TWEAK, TNF-related weak inducer of apoptosis; UCP, uncoupling protein.

As previously stated, other important pathways are involved in cachexia-associated muscle loss. Among these, pathways of autophagy certainly represent other important and widely activated mechanisms that underly protein breakdown in CC. However, such pathways are far less characterized than those related to ubiquitin-proteasome pathway. Some studies showed that markers of autophagy like Beclin-1, (an indicator of autophagy induction), p62 (a marker of lysosomal degradation) and the two forms of microtubule-associated proteins 1A/1B light chain 3B protein (LC3B-I and LC3B-II, used to measure autophagosome abundance) are overexpressed in SM of tumor-bearing animals and cancer patients (117–120). However, in spite of the increased autophagic production and activity, the findings gained so far showed impairment of autophagosome clearance in the muscle of cancer hosts, suggesting that the process of lysosomal degradation does not come to complete cargo degradation (117, 119, 120). A further interesting finding was the phosphorylation and subsequent activation of Unc-51 like autophagy activating kinase 1 (ULK1), a driver of autophagy, in SMs of cachectic mice (121). Such an event was suggested to be mediated by upstream activation of p38, which was previously reported to cause activation of C/EBPβ. The molecular pathways involved in SM atrophy are depicted in Figure 1.

### Dysregulated Pathways in AT

To date, knowledge about pathways involved in loss of AT under cachectic conditions is markedly less detailed than that available for loss of SM. However, it is now widely recognized that excessive degradation of fatty acids and energetic imbalance in AT can contribute to the destructive impact of cachexia on tumor hosts.

Under physiological conditions, AT lipolysis is an essential catabolic process that provides lipids and energy to tissues and organs following opportune stimulations. In particular, this process occurs after interaction between certain molecules (including β-adrenergic neurotransmitters) and their cognate G-protein-coupled receptors (GPCRs) expressed in adipocytes. Downstream of this event, a number of adenosine triphosphate (ATP) molecules are converted to cyclic adenosine monophosphate (cAMP) by GPCRs-stimulated adenylyl cyclases. Increased levels of cytosolic cAMP eventually lead to activation of protein kinase A (PKA) by phosphorylation, which in turn leads to phosphorylation of both HSL and perilipin-1, a protein acting as a protective coat for lipid droplets. This implies activation of HSL and its translocation to the surface of lipid droplets, resulting in greater access to triglycerides and enhanced lipolysis (12, 13). ATGL represents another key point of the lipolytic process, since its enzyme kinetics determines the rate limiting of triglycerides catabolism. Specifically, ATGL is mainly responsible for the first step of triglycerides hydrolyzation, which leads to the release of a single fatty acid and diacylglycerol (DAG). The other two steps of lipolysis are then completed by HSL and monoacylglycerol lipase (MGL), respectively (12).

Under conditions of chronic systemic inflammation, several events are known to occur affecting the AT metabolism, including suppression of appetite, enhancement of lipolysis and inhibition of LPL (122). The latter, in particular, has a dual function, being involved in receptor-mediated lipoprotein uptake and degradation, and hydrolysis of serum triglycerides in non-esterified fatty acids and 2-monoacylglycerol for tissue utilization. LPL deficiency leads to hypertriglyceridemia, while its upregulation causes insulin resistance and can promote obesity (123). Nonetheless, results from some studies clearly indicate that depletion of triglycerides in cachectic individuals is not due to impairments in LPL activity nor in lipogenesis, but depends on an increase in lipolysis, confirmed by high expression rates of HSL (124, 125).

Among procachectic cytokines, TNFα was shown to cause increased levels of phosphorylated HSL in adipocytes through activation of ERK, downstream of the MAPK signaling. Importantly, ERK-mediated activation occurred by phosphorylation on Ser 600 of HSL, which corresponds to the phosphosite targeted by cAMP-activated PKA (126). Instead, TNFα was not reported to induce any change in expression levels of HSL. Similarly, to TNFα, ZAG was demonstrated to cause hyperactivation of HSL by leading to increases in cAMP levels and subsequent activation of adenylyl cyclase in a dose dependent manner (93, 127). Moreover, differently from TNFα, ZAG was reported to induce upregulation of expression of Gαs (a G-protein subunit involved in activation of adenylate cyclase pathway) and HSL, and downregulation of expression of Gαi (a G-protein subunit involved in inhibition of adenylate cyclase pathway) (128). Interestingly, some experimental data suggested that ZAG expression in adipocytes might be negatively regulated by TNFα (92).

**Figure 2:**
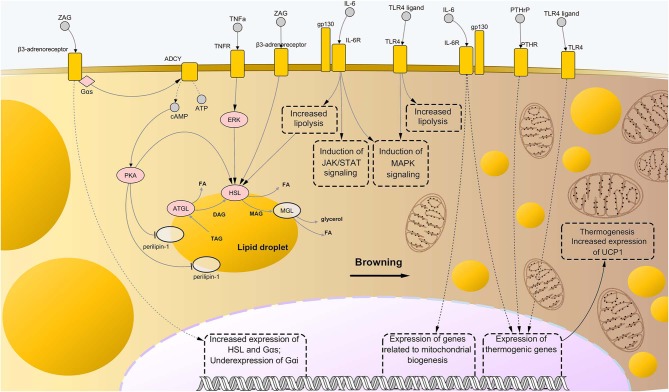
Molecular mechanisms driving adipose tissue loss and remodeling during cancer cachexia. Similarly to the case of skeletal muscle wasting, the combination of abnormally activated pathways including β-adrenergic signaling, cytokine- and toll-like receptor (TLR)-mediated inflammation, and parathyroid-related peptide (PTHrP) stimulation, leads to enhanced lipolysis and thermogenesis of the AT in cancer cachexia. In particular, binding of interleukin-6 (IL-6) and tumor necrosis factor alpha (TNFα) to their respective membrane receptors induce high phosphorylation levels of enzymes involved in catabolism of triglycerides, i.e., adipose triglyceride lipase (ATGL) and hormone-sensitive lipase (HSL), implying higher rates of their enzyme activity. Interaction between zinc-α2-glycoprotein (ZAG) and beta-3 adrenergic receptor is known to induce even stronger lipolytic effects than those mediated by IL-6 and TNFα, partly due to induction of expression of lipolytic genes (including G proteins of the type Gαs) and suppression of expression of Gαs, an inhibitor of β-adrenergic signaling. Moreover, ZAG promotes phosphorylation of perilipin-1, which results in wider exposition of lipid droplets to the catabolic action of ATGL, HSL, and monoglyceride lipase (MGL). A role in enhanced lipolysis during cancer cachexia has been demonstrated also for TLR4, although precise mechanisms remained largely elusive. Meanwhile, such pattern recognition receptor seems to exert an important role during cachexia-driven white adipose tissue browning, along with IL-6 and PTHrP, promoting expression of thermogenic genes, such as uncoupling protein 1 (UCP1), and favoring mitochondrial biogenesis. In the present illustration, the process of browning proceeds from the left to the right side of the figure, with the left side presenting bigger lipid droplets and almost mitochondria, while the right side presenting a large number of small, sparse lipid droplets and mitochondria. Upregulated nodes are colored in pink and connected with other nodes through continuous edges. Non-deregulated nodes are represented as semi-transparent nodes. Dashed edges represent connections between transcriptional/co-transcriptional factors and gene expression. ADCY, adenylate cyclases; ATP, adenosine triphosphate; cAMP, cyclic adenosine monophosphate; DAG, diglyceride; FA, fatty acid; gp130, glycoprotein-130 IL-6R, IL-6 receptor; JAK, Janus kinase; MAG, monoglyceride; MAPK, mitogen-activated protein kinase; PTHR, parathyroid receptor; STAT, signal transducer and activator of transcription; TAG, triglyceride; TNFR, tumor necrosis factor receptor.

Further evidence about involvement of procachectic cytokines in fat loss during cachexia was found for IL-6. High levels of this cytokine in the plasma of cachectic mice correlated with enhanced AT lipolysis and increased levels of circulating free fatty acids. These changes were shown to be associated with activation of IL-6 signaling in WAT, which caused downstream activation of STAT3 and p38 (11, 129). All the above mentioned pathways are represented in Figure 2.

### Enhanced Thermogenesis During CC

As outlined above, most of cachectic cancer patients experience involuntary excessive energy expenditure. A considerable fraction of this phenomenon has been attributed to aberrant activation of thermogenesis, which, in general, involves the interplay of two main actors, i.e., SM and the brown adipose tissue (BAT) (130). In both cases, this event seems to depend on expression of high amounts of uncoupling proteins (UCPs). UCPs are located on the inner membrane of mitochondria and redirect them toward heat generation instead of ATP synthesis (130). In particular, UCP1 seems to be prevalent in BAT, while UCP2 and UCP3 would be primarily expressed in SM (131, 132).

Browning of white adipocytes is the process by which adipocytes of the WAT are transformed into beige adipocites, consequently to endocrine, paracrine, or autocrine stimulation. This phenotypic transition seems to occur during the initial stages of CC, preceding SM atrophy (133). Several reports have argued that WAT browning mainly relies on increased expression of UCP1 in mitochondria of white adipocytes [e.g., (134)]. Nonetheless, some recent studies have remarked the non-consistency of these results for certain models of experimental cachexia and questioned the real importance of UCP1 in such a context. A possible key role was suggested for the transcriptional coactivator cell death inducing DFFA-like effector A (CIDEA), which is highly expressed in WAT and has a role in lipid metabolism (2). However, this topic has remained controversial.

Both PTHrP and IL-6 were reported to drive the expression of thermogenic genes *in vivo*, including UCP1, CIDEA, and PGC1α (10, 90). Consistent with this, neutralization of PTHrP or IL-6 in cachectic mice prevented WAT browning, rescued the cachectic phenotype and blocked the increased expression of UCP1 in subcutaneous AT of C26-bearing mice (10, 11). Moreover, silencing of IL-6 reversed the enhanced mitochondrial respiration in WAT of tumor-bearing mice toward the normal condition (11). However, it remained to understand whether these two factors induced thermogenesis by direct or indirect action on WAT/BAT.

Concerning PTHrP, the evidence that knockout of PTHR in AT blocked adipose browning and wasting provided a strong validation of direct action of PTHrP on both WAT and BAT (90). Surprisingly, the same study demonstrated that knockdown of PTHR in AT also prevented lean mass depletion and improved muscle strength. This raised the question whether PTHrP exerts a direct action even toward SM or not. Indeed, besides the well-known cross-talk between AT and SM (2, 135), a direct effect of PTHrP on myocytes could be also speculated. In fact, a previous report demonstrated that PTH1R expression is required for myocyte differentiation and is highly expressed by CD34- or PAX7-positive myosatellite cells in both mice and humans (136). In the same study, it was shown that myotubes also express PTH1R, albeit to a much lesser extent than differentiating myoblasts (136).

Regarding the role of IL-6 in WAT browning, elegant experiments carried on an *in vitro* model of pre-brown adipocytes demonstrated a direct, though modest, effect of IL-6 on UCP1 expression (11). Nonetheless, mechanistic insights regarding this aspect remained elusive. Indeed, IL-6 was suggested to mediate thermogenic effects on BAT through activation of p38, which is known to induce increased mitochondrial biogenesis and expression of thermogenic genes (137, 138). Another possible mechanism would involve the IL-6-mediated activation of AMP-activated protein kinase (AMPK), which in turn would cause an increase in hydrolase activity of ATGL and inactivation of mTORC1 (129). However, the dominant idea is that IL-6-mediated enhancement of thermogenesis and lipolysis is the outcome of both direct and indirect actions of this cytokine on BAT and WAT (139–142).

A role in increased lipolysis and WAT browning was recently demonstrated for TLR4 as well (74). Precisely, LLC-bearing mice showed enhanced lipogenic activity and AT remodeling, while cachectic mice knockout for TLR4 showed reduced thermogenic activity BAT, underexpression of UCP1, and lower phosphorylation levels of p38 compared to the former group (74). This may suggest a potential connection between TLR4, MAPK signaling (70) and transcription of UCP1. Pathways related to WAT browning are represented in Figure 2.

### Discrepancies: From the Laboratory to the Clinical Practice

In spite of the considerable knowledge we have lastly gained regarding CC, the clinical practice still lacks standard diagnostic criteria and treatment guidelines. One of the main reason for such difficulties certainly reside in the multifactorial nature of this syndrome, and thus in its intrinsic complexity. On the other hand, however, the scarceness of experimental models of CC capable to accurately recapitulate the features of human CC in various tumor types has represented a major limitation in relation to this issue.

Most preclinical studies conducted so far have been mainly based on exploitation of few ascertained animal models of cachexia-inducing tumors, including LLC, C26 and Yoshida ascites hepatoma (YAH 130) (143). These were chosen and maintained as general models of CC owing to the broad documentation in their regard. However, a lot of skepticism have been lastly raised about their actual potential to be employed as reliable surrogates of human CC. In particular, it is now becoming predominant the opinion that the failure of most anti-cachexia therapeutics to reach primary endpoints during clinical trials (see the next section) is at least in part attributable to the limitations imposed by the use of inappropriate experimental models (143). In line with this reasoning, a number of recent studies have demonstrated the relative inconsistency of such models, since their molecular characterization at the tissue level revealed quite different patterns of gene expression between animal models and human patients [e.g., (144, 145)].

In this context, genetically engineered mouse (GEM) models of CC are being evaluated as a relevant solution to overcome such restrictions. GEM models currently tested for preclinical studies avail of the tamoxifen-inducible conditional genetic mutation system Cre-ER (146). Adenovirus-containing Cre-ER encodes for a fusion protein in which a mutated hormone-binding domain of the estrogen receptor (ER^T^ or ER^T2^) is fused to Cre, a bacterial site-specific recombinases that catalyzes recombination between well-defined “*loxP*” sites flanking the gene of interest (146–148). As this system relies upon the presence of tamoxifen-inducible promoters, it allows the spatio-temporal control of Cre expression, with the subsequent deletion of genes flanked by *loxP* sites. In contrast with tumors developed by traditional CC models, tumors arising in GEM models closely mimic the histopathological and molecular features of their human counterparts and spontaneously progress toward metastases and cachexia.

A valuable GEM model of CC is that engineered by Talbert et al. (144), which was proved to closely resemble the typical features reported for cachexia induced by pancreatic ductal adenocarcinoma (PDA) in human patients. The mouse model, named KPP (Kras^+/G12D^, Ptf1a^+/ER−Cre^, Pten^f/f^), exhibited progressive wasting of SM, heart and AT mass, similar to that experienced by humans, and gene ontology of its SM-derived mRNAs resembled that of PDA patients (144). By contrast, this model displayed loss of normal pancreatic parenchyma, which could significantly contribute to weight loss in KPP mice. Furthermore, it is likely that such a model is selective for PDA, and thus not suitable for studying cachexia associated to other tumor types (144). In the same study, the GEM model KPC (Kras^+/LSL−G12D^, Trp53^+/R270H^, Pdx1^+/Cre^) was reevaluated. Indeed, KPC is a GEM model of PDA (149) that has been recently considered a reliable model of cachexia by a number of studies [e.g., (11, 150, 151)]. Nonetheless, Talbert et al. demonstrated that changes in size and tissue mass of KPC mice did not correlate with PDA progression, and that patterns of gene expression in their SM did not resemble that of PDA patients (144). Thus, the KPP genotype appears to be more robust for research on CC compared with the KPC model. Further examples of potentially useful GEM for research on CC are the KL model (KRAS^G12D/+^; LKB1^f/f^), recently used to study the metabolic profile of advanced non-small cell lung cancer (NSCLC) (152), and the lately developed KPC:APC model (APC^f/f^; KRAS^+/f^; CDX2-Cre-ER^T2^), which aims to recapitulate features of human colorectal carcinoma (CRC) with mutated KRAS and leads to development of CC (153).

Orthotopic models of patient-derived cancer cells (PDCC) may represent another valuable option for research on CC. Here, tumor cells derived from human patients are seeded into the corresponding tissue of immunocompromised animal models. This has the obvious advantage of allowing the study of organ-specific tumor microenvironment with increased accuracy and higher genetic heterogeneity and stability compared to the traditional xenograft models, similarly to GEM models. In particular, this was demonstrated for both orthotopic and subcutaneous engraftment of patient-derived pancreatic tumor (154, 155). However, some data suggested that orthotopic engraftment of pancreatic tumor cells within mice's pancreas could induce systemic inflammation distinct from that observed in subcutaneous engraftments, leading toward more severe SM wasting [e.g., (154)].

One limitation of orthotopic models of CC is that here engrafted PDCC maintains their metastatic potential (154, 156), thus preventing researchers to study stages related to development and progression of cachexia. Moreover, lean mass loss in these models may be the result of post-surgical inflammation instead of tumor-induced tissue loss. Yet, the exploitation of immunocompromised mice for generating orthotopic models may fail in reproducing the immune profile of CC (144). The latter limitation, however, can be overcome by employing syngeneic mouse models of GEM-derived tumors, i.e., models in which immune-competent mice are implanted with tumor cells derived from GEM presenting the same genetic background [e.g., (157)].

## Current Unimodal Treatment Options for CC

There is a wide range of treatment options currently available for CC. Nutritional support is very important in cancer patients, as their food intake is often reduced due to symptoms like anorexia caused by systemic inflammation, nausea, vomiting, and mucositis (158). For these reasons, as soon as patients are diagnosed with cancer, they should be nutritionally monitored and receive both nutritional and metabolic support (159). Meanwhile, since systemic inflammation is a hallmark of CC, the pharmacological targeting of individual proinflammatory cytokines or that of their cognate receptors has been considered as a potential powerful approach. Also, several hormones and hormone-derived compounds known to stimulate appetite and to induce weight gain have been tested. Nonetheless, most of the current trialed agents has not received the FDA (Food and Drug Administration) approval since either no substantial benefits or manifestation of important side effects were observed throughout Phase I/III of clinical trials (143, 160) (Table 1).

**Table 1:** Table summarizing unimodal treatment options mentioned in this review.

**Drug name**	**Drug class**	**Molecule type**	**Disease**	**Status**	**Positive outcomes**	**Adverse effects/Difficulties**	**References**
Etanercept	Anti-TNFα therapy	TNFα receptor-blocker	Cancer (other than brain cancer)	Phase III RCT (placebo)	–	Induced negligible weight gain; did not improve median survival; caused higher rates of neurotoxicity.	NCT00046904 (161)
Thalidomide	Anti-TNFα therapy	Synthetic derivative of glutamic acid	Pancreatic cancer	RCT (placebo)	Attenuated weight and lean body mass losses	No significant improvements of QoL	(162)
Thalidomide	Anti-TNFα therapy	Synthetic derivative of glutamic acid	Cancer	Phase III RCT	Significant decrease in levels of circulating IL-6; did not cause worsening of life	Lack of satisfactory documentation	(163)
Thalidomide	Anti-TNFα therapy	Synthetic derivative of glutamic acid	Cancer	RCT (placebo)	Slight decrease in levels of circulating IL-6 and TNFα	No significant benefits compared with placebo; possible risk of treatment-related mortality	(164)
Pentoxifylline	Anti-TNFα therapy	Methylxanthine derivative	Cancer (other than brain cancer)	RCT (placebo)	–	No differences in QoL and possible worsening of life after 4 weeks of treatment	(165)
Pentoxifylline	Anti-TNFα therapy	Methylxanthine derivative	Cancer	RCT (placebo)	No toxicity	Did not improve appetite nor significant weight gain	(166)
Infliximab	Anti-TNFα therapy	Chimeric IgG1k monoclonal antibody	NSCLC	Phase III RCT (placebo)/ docetaxel	–	Did not induced weight gaining; caused increased fatigue and inferior global QoL	NCT00040885 (167)
Clazakizumab	Anti-IL6 therapy	Humanized anti-IL-6 monoclonal antibody	Cancer	Phase I CT	No apparent toxicity; increased hemoglobin and albumin levels; reduced fatigue	Lack of satisfactory documentation	(168)
Clazakizumab	Anti-IL6 therapy	Humanized anti-IL-6 monoclonal antibody	NSCLC	Phase II RCT	Generally well tolerated; improved the lung symptom score; attenuated lean mass loss; reversed fatigue	May cause rectal hemorrhage or treatment-related mortality in a minority of patients; inconclusive in terms of clinical management	NCT00866970 (169)
Selumetinib	Anti-IL6 therapy	MEK1/2 inhibitor	Biliary cancer	Phase II CT	Overall acceptable tolerability; induced significant weight gaining	Induced low-grade adverse events including rush and xerostomia; may worsen fatigue in a minority of patients	NCT00553332 (170)
Selumetinib	Anti-IL6 therapy	MEK1/2 inhibitor	Cholangiocarcinoma	Phase II CT	Induced significant gaining of lean body mass	Lack of satisfactory documentation	(22)
Ruxolitinib	Anti-IL6 therapy	JAK1/2 inhibitor	Cancer	Phase II CT	–	The study was terminated due to poor recruiting	NCT02072057
Xilonix	Anti-IL-1α therapy	IL-1α-specific humanized monoclonal antibody	Cancer	Phase I CT	Well tolerated by all participants, no dose-limiting toxicities were reported	Caused proteinuria, anemia, nausea and fatigue in a minor fraction of patients	NCT01021072 (171)
Xilonix	Anti-IL-1α therapy	IL-1α-specific humanized monoclonal antibody	CRC	Phase III RCT (placebo)	Prevented alteration of body composition and improved control of thrombocytosis	Caused proteinuria, anemia, increased concentration of alkaline phosphatase and aspartate aminotransferase and fatigue in a minor fraction of patients	NCT01767857 (172)
Xilonix	Anti-IL-1α therapy	IL-1α-specific humanized monoclonal antibody	CRC	Phase III CT	–	The study was stopped as it crossed the prospective futility boundary of primary endpoint	NCT01767857
Celecoxib	NSAID	Cyclooxygenase-2 (COX-2) inhibitor	Cancer	Phase II RCT (placebo)	Significantly improved BMI and QoL; moderate decrease of IL-6 levels after 3 weeks of treatment	–	(173)
Celecoxib	NSAID	Cyclooxygenase-2 (COX-2) inhibitor	Cancer	Phase II CT	Significantly improved BMI and QoL; moderate decrease of TNFα levels; increased handgrip strength and improved performance status	–	(174)
LY2495655	Mstn inhibition	Monoclonal antibody to Mstn	(1) Cancer (2) Healthy	(1) Phase I RCT (placebo) (2) Phase I CT	Well tolerated; no dose-limiting toxicities were reported; increase in thigh muscle volume; consistent increases in handgrip strength observed at doses ≥21 mg; improvement in functional measures	No clear trend in dose-dependent efficacy	NCT01524224 (175)
LY2495655	Mstn inhibition	Monoclonal antibody to Mstn	Pancreatic cancer	Phase II RCT (placebo) + standard chemotherapy	–	No significant improvements in muscle volume; pre-cachectic patients were more responsive than cachectic patients; trend toward poorer overall survival in treated patients vs. placebo	NCT01505530 (176)
Bimagrumab	Mstn inhibition	Human monoclonal anti-ActRII antibody	NSCLC and Pancreatic adenocarcinoma	Phase II RCT (placebo)	Significant increase in lean body mass and thigh muscle volume	Significant decrease in total body weight	NCT01433263
AMG 745	Mstn inhibition	Fc fusion peptibody	Prostate cancer	Phase I RCT (placebo)	Generally well tolerated; increased lean body mass	Slight decrease in fat mass; may cause adverse events including diarrhea and fatigue	NCT00975104 (177)
Megestrol acetate (FDA approved)	Appetite stimulant	Progesteron derivative	Several cancer types	Summary of 35 CT	Improvement of appetite and increased caloric intake, weight gain and nutritional status; improvement of QoL; downregulation of proinflammatory cytokines or that of their cognate receptors	More than 40 side effects including edema, thromboembolitic episodes and treatment-related death	Summary of 35 clinical trials (178)
Medroxyp rogesterone acetate (FDA approved)	Appetite stimulant	Progesteron derivative	Several cancer types	Summary of most relevant CT	Improved anorexia and QoL parameters; impaired synthesis and release of IL-6, IL-1, and TNFα	Weight gain was due to increased body fat mass rather than lean body mass	Summary of most relevant clinical trials (179)
Ghrelin	Orexigenic mediator	Hormone	Esophageal cancer	Phase II RCT (placebo) + cisplatin-based neoadjuvant chemotherapy	Increased food consumption and weight gain; reduced nausea and anorexia related to chemotherapy	–	(180)
Anamorelin HCl	Orexigenic mediator	Ghrelin Receptor agonist	NSCLC	Phase III	Generally well tolerated; improved appetite; increased food intake, body weight and lean body mass	Caused hyperglycemia, nausea and gastrointestinal disorders in a minority of patients; no significant improvement of handgrip strength	NCT01387269 (181)
Anamorelin HCl	Orexigenic mediator	Ghrelin Receptor agonist	NSCLC	Phase III	Generally well tolerated; improved appetite; increased food intake, body weight and lean body mass	Caused hyperglycemia, nausea and gastrointestinal disorders in a minority of patients; no significant improvement of handgrip strength	NCT01387282 (181)
Anamorelin HCl	Orexigenic mediator	Ghrelin Receptor agonist	NSCLC	Phase III	Generally well tolerated; improved appetite; increased food intake, body weight and lean body mass	Caused hyperglycemia, nausea and gastrointestinal disorders in a minority of patients; no significant improvement of handgrip strength	NCT01395914 (182)
THC	Appetite stimulant and metabolism modulator	Endogenous agonist of CB1 and CB2 receptors	Cancer	Phase III CT	Well tolerated, no adverse effects	No significant improvements in appetite or QoL	(183)
THC	Appetite stimulant and metabolism modulator	Endogenous agonist of CB1 and CB2 receptors	Cancer	Phase III CT	Well tolerated, no adverse effects; significant increase in appetite and caloric intake; improved chemosensory perception and QoL	–	NCT00316563 (184)
THC	Appetite stimulant and metabolism modulator	Endogenous agonist of CB1 and CB2 receptors	Cancer	Pilot study	Well tolerated; significant increase in appetite and caloric intake; improved QoL; reversed fatigue	May induce dizziness or anxiety in a small fraction of patients	NCT02359123 (185)
Nabilone	Appetite stimulant and metabolism modulator	Synthetic analog of THC	NSCLC	Phase II RCT (placebo)	Increased appetite and caloric intake; improvement of QoL; attenuated pain and insomnia	–	NCT02802540 (186)
Erythropoietin	Anemia reversal	Hormone	Cancer	Randomized study	Reversed anemia; improved exercise ability and sense of well-being	No significant improvements in QoL; discrepancies between objective and subjective self-reported measures	(187)

*CT, clinical trial; RCT, randomized clinical trial*.

### TNFα, IL-6, and IL-1 Inhibitors

Because of their widely recognized role in induction of cachectigenic effects, TNFα, IL-6, and IL-1α had represented the ideal target of several anti-cachectic compounds. However, in the case of anti-TNFα therapy, neither the TNFα receptor–blocker Etanercept (161) nor the chimeric IgG1 kappa monoclonal antibody Infliximab (167) were able to prevent muscle atrophy or improve appetite in two randomized controlled trials of terminal cachectic patients. Moreover, Infliximab was found to increase fatigue and treatment-related mortality in administrated patients (167). Likewise, slight recovery of muscle mass accompanied by worsening of life or no significant beneficial effects were reported by five randomized clinical trials testing two pharmacological agents capable to downregulate expression of TNFα, namely Thalidomide (162–164) and Pentoxifylline (165, 166), respectively.

Anti-IL-6 and anti-IL-1α pharmacological agents showed promising results over the course of clinical trials; nonetheless, they were inconclusive in terms of clinical management of the disease. Specifically, in a Phase I clinical trial involving patients with advanced cancer, the humanized anti-IL-6 monoclonal antibody Clazakizumab was well tolerated, increased hemoglobin and albumin levels and reversed fatigue in treated patients (168). During the subsequent Phase II randomized controlled trial, administration of Clazakizumab to patients with NSCLC resulted in a slight lower degree of lean mass depletion (169). However, effects on SM mass were not considered satisfactory according to the acceptance criteria and further studies were required (188). Similarly, in Phase I and III clinical trials, the IL-1α-specific humanized monoclonal antibody Xilonix was found to prevent alteration of body composition and improve control of thrombocytosis in refractory cancer patients (171) and advanced CRC patients with cachexia (172), respectively. The most common adverse events observed at the end of these studies were proteinuria (171), anemia and increased concentration of both alkaline phosphatase and aspartate aminotransferase (172). A second phase III clinical trial attempting to further demonstrate the efficacy of Xilonix in advanced CRC patients (NCT01767857) was stopped at its early stage as the study crossed the prospective futility boundary of primary endpoint.

Indirect blockage of IL-6 action by pharmacological inhibition of the JAK/STAT3 signaling may represent another valid strategy to be persued. In preclinical studies, the allosteric inhibitor of MAPK/ERK kinase 1 (MEK1) and 2 (MEK2) Selumetinib was proved to exert tumor suppressive activity and prevent cancer-induced IL-6 production [e.g., (189)]. Yet, results from a Phase II trial conducted in patients with metastatic biliary cancers suggested that Selumetinib induced non-fluid weight gain in administrated patients (170). Thus, this drug was tested in a Phase II trial in patients with advanced cholangiocarcinoma, aiming to study its potential as modulator of IL-6/JAK/STAT3 signaling and mediator of SM anabolism (22). Despite results showed that Selumetinib effectively promoted SM gain in patients with cholangiocarcinoma, its actual relevance for CC remained to be demonstrated (22).

Similarly, the selective JAK1/2 inhibitor Ruxolitinib was trialed on patients with myelofibrosis and was found to reduce splenomegaly and disease-related symptoms, meanwhile inducing a significant increase in body weight (190). An open-label Phase II trial was then started in 2014 aiming to examine both safety and efficacy of Ruxolitinib as well as its influence on overall survival. However, enrollment of participants for this study did not succeed and the trial was unsuccessfully terminated at the beginning of 2019 due to poor recruiting (NCT02072057).

### Non-steroidal Anti-inflammatory Drugs

Non-steroidal anti-inflammatory drugs (NSAID) have been considered to counteract chronic inflammation in CC as well. In particular, Celecoxib, a cyclooxygenase-2 (COX-2) inhibitor, significantly improved QoL and BMI in cachectic patients vs. control (173). Another study reported significant increases of lean mass and improvements in handgrip strength and QoL in patients with advanced cancers administrated with Celecoxib (174). In addition, a significant decrease in TNFα levels was observed, and no particular toxic effects were reported (174). More recently, several trials have evaluated the effects of a combination of Celecoxib with other drugs on cachectic patients, including combination with L-carnitine [a nutritional supplement that improves the cachectic condition (191)] and combination of these two compounds plus Megestrol acetate (see below). These studies led to more effective improvements in lean mass, total physical activity and decreased sense of fatigue, without toxic effects (192).

### Myostatin Inhibitors

In recent times, the treatment of cachexia has been focused on myokines, which include cytokines and other proteins produced and secreted by SM cells (193, 194). Among these, Mstn can act as autocrine, paracrine or endocrine effector along with cytokines like IL-6, IL-8, and IL-15, and is responsible for immune and metabolic changes associated to exercise or stress (193–195). Thus, Mstn inhibitors have been considered promising tools for the treatment of cachexia. In 2012, two Phase I clinical trials under the same study evaluated the safety and tolerability of the humanized monoclonal antibody to myostatin LY2495655 in healthy subjects and patients with advanced cancer (175). According to the results obtained, the efficacy of LY2495655 was well demonstrated in the case of healthy volunteers, in which it caused an increase in thigh muscle volume. Increases in muscle volume were observed also in patients with advanced cancer, though only when the drug was administrated at relatively low doses (21 and 70-mg) without unusual safety concerns (175). In 2018, a Phase II trial was performed on a group of inoperable patients or with metastatic pancreatic cancer, who suffered from cachexia. Here, patients were treated with different doses of LY2495655, and the authors reported that pre-cachectic patients were more responsive to the treatment than cachectic patients (176). These results may suggest that treatment with LY2495655 should be better considered for prevention muscle loss rather than for reverting the process, although deeper investigation and additional functional readouts are required.

In 2014, one study described the properties of the human anti-ActRII antibody Bimagrumab (BYM338) (196). BYM338 prevented the binding of ligands to the receptors and hence the activation of signaling pathway downstream of ActRII. *In vivo*, administration of bimagrumab resulted in significant SM hypertrophy and increased muscle fiber diameter. Furthermore, it protected muscles from glucocorticoid-induced atrophy and weakness through the impairment of muscle and tetanic force losses (196). Lastly, Novartis Pharmaceuticals completed a randomized control trial of BYM338 for treatment of CC associated with pancreatic adenocarcinoma and NSCLC. Patients administrated with Bimagrumab displayed significant increases in lean body mass and thigh muscle volume, yet also had decrement in total body weight (NCT01433263).

Another myostatin inhibitor is AMG 745, a peptibody resulted from the fusion of a human N-terminal Fc region of an immunoglobulin and the C-terminus of a myostatin-neutralizing peptide. AMG 745 has been successfully tested in various murine models including C26 tumor-bearing mice, where it increased SM mass, body weight and strength with respect to the control group (177). In patients undergoing androgen deprivation as a consequence of non-metastatic prostate cancer, AMG 745 was reported to improve the lower-extremity muscle mass (177).

### Metabolism and Appetite Modulators

One of the first pharmacological options proposed for the treatment of CC include Megestrol acetate, an appetite stimulant derived from progesterone capable to improve caloric intake and nutritional status. Albeit its precise mechanism of action still remains undetermined, it has been proven that this hormone-derivated drug exerts an anti-inflammatory action, as it has the ability to downregulate proinflammatory cytokines levels or that of their cognate receptors (197, 198).

After being tested in several trials, Megestrol acetate was reported to improve cancer patients' appetite, QoL and weight gain compared to placebo group, but not when compared to patients treated with other drugs (178). For this reason, the FDA approved it in 1993 for the treatment of anorexia, cachexia and unexplained weight loss in patients with AIDS (178). Nonetheless, Megestrol acetate was found to be responsible of more than 40 side effects, including odema, thromboembolic episodes and death (178). Yet, a case study about a 65 year-old man who suffered from metastatic renal cell carcinoma revealed that Megestrol acetate can induce adrenal insufficiency (199). More recently, the efficacy of the combination of Megestrol acetate plus Thalidomide was tested in cachectic cancer patients compared to the treatment with Megestrol acetate alone (200). At the end of the study, results revealed that the combination of these two drugs was more effective than the treatment with Megestrol acetate alone, as patients showed statistically significant increase in body weight, appetite, QoL and grip strength, as well as significant decrease of IL-6 and TNFα levels (200).

Medroxyprogesterone acetate is another progesterone derivative taken into consideration for the clinical treatment of cancer-related anorexia/cachexia syndrome. At the same extent of Megestrol acecate, Medroxyprogesterone has been shown to impair the synthesis and release of several procachectic cytokines, in particular IL-6, TNFα, and IL-1 (179, 197). In placebo-controlled studies, it was reported that Medroxyprogesterone generally improved anorexia, QoL and body weight gain, although the latter was caused by an increase in AT rather than lean mass.

Due to his orexigenic properties, ghrelin has been evaluated as palliative remedy in CC. Ghrelin is mainly produced and secreted by the stomach and, to a lesser extent, by other organs such as pancreas, lung, liver, muscle, and AT (201). Ghrelin mediates multiple pathways involved in the regulation of body weight, body composition, energy storage and appetite, and regulates energy expenditure (202). In particular, it has been demonstrated that this hormone causes an increase in food consumption in both rodents and human patients with cancer, leading to significant weight gain [e.g., (180, 202)]. Moreover, patients who received ghrelin manifested less adverse effects during chemotherapy in terms of nausea and anorexia in respect to the control group (180). Thus, ghrelin is considered a potential remedy for the management of CC. However, an apparent contradictory observation was reported by some studies. In fact, cachectic murine models and cancer patients with cachexia both usually presented hyper-ghrelinemia, though paradoxically showing loss of appetite. This is probably due to grelin's short half-life, which is nearly 30 min (201, 203). In order to solve this issue, more stable ghrelin analogs have been developed as potential therapeutic agents. Among them, Anamorelin HCl has a half-life corresponding to nearly 7 h and is considered a valid agonist of the ghrelin receptor, as it displays significant appetite-enhancing activity and increases food intake and body weight in rats in a dose-dependent manner (204).

Anamorelin was tested on a group of volunteers in a randomized Phase I clinical trial. At the end of the trial, subjects who underwent the treatment displayed a significant dose-related weight gaining compared to the placebo group, with no particular adverse effects (205). Anamorelin has also been tested in several Phase II trials, with patients suffering from different types of solid and blood tumors, showing safety and efficacy (206). Notoriously, Anamorelin has been widely tested on patients with NSCLC during the ROMANA international clinical trials. ROMANA 1 and ROMANA 2 are two randomized Phase III trials performed on cachectic patients with incurable stage III/IV NSCLC for 12 weeks (181). In both studies, the Functional Assessment of Anorexia/Cachexia Therapy (FAACT) and the fatigue domain of the Functional Assessment of Chronic Illness Therapy-Fatigue (FACIT-F) showed an improvement in patient conditions, with an increase in body weight compared to the placebo group (181). Across the groups, Anamorelin treatment was well tolerated and caused general improvement of anorexia-cachexia symptoms, with the exception of some low incidence adverse events such as grade 1-2 hyperglycemia, nausea and gastrointestinal disorders (181).

A subgroup of patients who completed both trials were successively recruited for the ROMANA 3 study. Patients continued to receive Anamorelin at 100 mg a day or placebo for additional 12 weeks (182). Overall, the treatment was still well tolerated and improvement in body weight and anorexia-cachexia symptoms were significantly increased from baseline of original trials at all time points vs. placebo (182). Nonetheless, the drug has not been approved by FDA since the ROMANA trial did not demonstrated significant benefit handgrip strength, which is one primary outcome required for drug approval.

Further palliative care may be provided by endocannabinoids, i.e., endogenous agonists of the cannabinoid receptors CB1 and CB2. These two receptors are expressed at different levels in both neuronal and non-neuronal tissues, including the gastrointestinal system, SM and AT (207). To date, the endocannabinoid system is known to regulate appetite by differential activation of two brain sites, namely the limbic system and hypothalamus; moreover, it exerts an influence on intestinal and AT functions (207). Among endocannabinoids, Δ-9-tetrahydrocannabinol (THC) was tested as appetite stimulant in a Phase III clinical trial. Although the drug was well tolerated throughout the study, no significant differences were observed in terms of appetite or improvement in QoL between treated patients and the placebo group (183). However, more promising results were obtained by a later trial (184) and a recent pilot study (185) for the same drug. More recently, the synthetic analog of THC nabilone was reported to significantly increase appetite in cachectic NSCLC patients during a Phase II trial, with subsequent increase in patients' caloric intake. The same study reported a significant improvement in patients' QoL and no adverse effects (186).

### Erythropoietin

Anemia is an additional feature displayed by cancer patients who suffer from cachexia, and contributes to weight loss and various metabolic alterations (187, 208). Thus, erythropoietin was tested in unselected cancer patients on palliative care. Patients treated with erythropoietin manifested a number of clinical benefits including improved exercise ability and sense of well-being (187). In mouse models of CC, it was shown that treatment with erythropoietin can counteract AT wasting and increase the lipogenic rate through the activation of erythropoietin receptor (EpoR) (208). Moreover, erythropoietin administration improved the survival of cachectic mice along with their exercise capacity, the latter being a consequence of increased erythrocyte count (209).

### New Upcoming Potential Approaches

Results from latest experimental studies let emerge new potential therapeutics for the clinical management of CC, relying on different strategies than those previously followed (Table 2). For instance, the targeting of endogenous AT and energy metabolism modulators underlying CC may represent a valuable therapeutic option, as in the case of anti-PTHrP antibody (10). Another example is represented by the pharmacological inhibition of fatty acid oxidation by Etoxomir, a carnitine palmitoyltransferase-1-selective inhibitor. In a preclinical setting, Etoxomir was shown to effectively rescue human myotubes *in vitro* as well as muscle mass and body weight of cachectic mice (210). Yet, Metformin was shown to prevent WAT browning by increasing PP2A activity, with subsequent dephosphorylation of acetyl-CoA carboxylase and HSL (211). Targeting of hepatic peroxisome proliferator-activated receptor-α (PPARα) by PPARα agonists has also show a considerable potential. In particular, administration of the PPARα agonist fenofibrate prevented loss of SM mass and body weight in KL mice (152). Moreover, this treatment was capable to reduce elevated glucocorticoid levels in the serum of KL mice, which result from IL-6-induced suppression of hepatic ketogenesis and cause impairment of intratumoral immunity (213).

**Table 2:** Table summarizing emergent unimodal treatment options for CC recently tested in preclinical studies.

**Drug name**	**Drug class**	**Molecule type**	**Disease**	**Status**	**Positive outcomes**	**Adverse effects/Difficulties**	**References**
anti-PTHrP	PTHrP inhibitor	Monoclonal neutralizing antibody against PTHrP	LLC murine model	Preclinical study	Prevented weight loss, AT browning and preserved lean body mass; improved physical activity; suppressed thermogenic gene expression	–	(10)
Etoxomir	Fatty acid oxidation blocker	Carnitine palmitoyltransferase-1-selective inhibitor	Stable murine models of human cancer-induced CC (by injection)	Preclinical study	Rescued muscle mass and body weight of cachectic mice	–	(210)
Metformin	Inhibitor of complex I of the electron transport chain	Biguanide compound	Murine burn model	Preclinical study	Prevented WAT browning by increasing PP2A activity	–	(211)
Fenofibrate	PPARα agonist	Fibric acid derivative	GEM model of NSCLC	Preclinical study	Prevented loss of SM mass and body weight; reduced glucocorticoid levels in mice serum	–	(152)
AR-42	Epigenetic modulator of gene expression	I/IIB HDAC inhibitor	LLC and C26 murine model	Preclinical study	Preserved muscle and fat mass; prolonged survival, reduced splenomegaly, reduced levels of Atrogin-1, MuRF1 and pro-inflammatory cytokines	The treatment showed distinct efficacy between the two models of CC	(212)
Atorvastatin	TLR inhibitor	HMG-CoA reductase enzyme inhibitor	LLC murine model	Preclinical study	Increased body weight; decreased tumor mass; attenuated AT remodeling; decreased levels of pro-inflammatory cytokines; prolonged survival	–	(74)
IMO-8503	TLR inhibitor	Immune modulatory oligonucleotide	LLC murine model	Preclinical study	Attenuated loss of lean mass; decreased levels of Pax7; prevented caspase-3 and PARP cleavage in SM	Caused toxic effects when administered at a concentration ≥ 15 mmol/L	(75)
Resiquimod (R848)	Topical TLR7/8 agonist	Imidazoquinoline compound	KPC-derived PDA syngeneic murine model	Preclinical study	Impaired expression of PD-1 on T cells; enhancement of CD8+ T-cell cytotoxic response; decreased tumor burden and to improvement of CC features	Initial or prolonged hypophagia and weight loss; may cause initial treatment-related decreases in locomotion	(157)

Further potential drugs that have recently been considered for clinical management of CC belong to the family of histone deacetylase (HDAC) inhibitors. Experimental evidences demonstrated that HDAC1, a class I HDACs family member, is required for muscle atrophy and contractile dysfunction caused by SM disuse and nutrient deprivation, although such mechanism can occur also independently from loss of SM (214). In line with this, a comprehensive study was performed in 2015 showing that AR-42, a novel class I/IIB HDAC inhibitor, displayed anti-cachectic activity in two independent murine models of CC (212). In both models, AR-42 reduced expression of Atrogin-1 and MuRF1 and production of certain proinflammatory cytokines, while significantly improved typical CC features such as body weight loss, reduced survival, muscle and AT loss, reduction of muscle strength, and muscle fiber size (212).

Finally, results obtained from preclinical studies investigating the effects of TLR inhibitors in CC experimental models have raised expectations for the future employment of these drugs in clinical trials (see below), although further investigations on more reliable models of CC are certainly required.

### TLR Inhibitors: Novel Therapeutics for Efficient CC Management?

Involvement of TLRs in muscle atrophy (TLR4 and TLR7/8) and AT remodeling (TLR4) has been highlighted by several *in vitro* and *in vivo* studies. These findings led to the development of efficient TLR inhibitors and antagonists, which are currently being considered as part of a new therapeutic strategy against CC. Nonetheless, to date, only a small number of such pharmacological agents is available for clinical use (215).

3-hydroxy-3-methyl-glutaryl-CoA (HMG-CoA) reductase inhibitor, also known as atorvastatin, is a pharmacological inhibitor of TLR4 (74). Atorvastatin impairs the inflammatory pathways through the inhibition of NF-κB activation and downregulation of TLR4 and MyD88 gene expression (215, 216). This drug resulted to be a good candidate as potential anti-cachectic tool since in LLC-bearing mice it significantly prolonged animal survival, increased body weight, impaired AT atrophy and browning and tumor mass compared to untreated animals. Moreover, atorvastatin administration resulted in a decreased concentration of pro-inflammatory cytokines at the plasma level (74).

Several oligonucleotide-based antagonists have been developed to prevent endosomal TLR overactivation in patients with autoimmune diseases (215). Among these, IRS-954 and DV-1179 represent the first two oligonucleotide-based TLR antagonists tested throughout preclinical studies and Phase I/II clinical trials, respectively. However, in spite of the safety shown by these two drugs, none of them were approved for the clinical use due to the lack of adequate documentation (IRS-954) or failure to comply with primary endpoints (215). Few years later, other immune modulatory oligonucleotide (IMO) were developed by Idera Pharmaceuticals with the same goal. Among these, IMO-9200 and IMO-8400, two TLR7, 8 and 9-specific antagonists, were tested in clinical trials and the outcomes were more successful than those obtained for DV-1179 (215). However, none of these drugs had yet been considered for clinical management of CC.

In 2018, our group carried out an *in vitro* and *in vivo* study in order to test the ability of IMO-8503 to impair miR-21-5p-induced myoblast cell death (75). Results were very encouraging as IMO-8503 significantly inhibited apoptosis induced by lung and pancreatic cell-secreted miR-21-5p and miR-29a-3p on both immortalized and primary myocytes. The same results were obtained with myocytes exposed to cancer patients-derived microvesicles. *In vivo*, IMO-8503 strongly impaired several cachexia features, including the loss of lean mass in tumor-bearing mice (75).

Despite the potential of TLR inhibitors in the management of CC, it should be recalled that also TLR7 agonists have been explored as antitumor agents for treatment of advanced cancers, alone or as vaccine adjuvants. In particular, Imiquimod (R837), a topical TLR7 agonist, was approved by FDA for the treatment of patients with basal cell carcinoma and skin metastases [e.g., (217)]. Imiquimod is generally well-tolerated, with only a minority of patients reporting grade I or II side effects, and lead to significant increases in the activity of both CD8+ and CD4+ T cells. INFα levels were markedly increased in tumor samples from treated patients, while only slight increases were reported for INFγ levels (217). Similarly, recent studies have suggested that Resiquimod (R848), a more potent counterpart of Imiquimod, may be beneficial for cancer patients suffering of cachexia (157). R848 is a TLR7 agonist that was proved to cause lower expression of programmed cell death protein 1 (PD-1) on T cells and enhancement of CD8+ T-cell cytotoxic response in cancer through an IL-12-mediated mechanism (218). R848 was reported to be able to lower tumor burden and improve cachexia manifestations in experimental models of pancreatic ductal adenocarcinoma (157).

## Conclusions

Over the last two decades, the study of CC has provided new insights that allowed a better understanding of the multiple mechanisms regulating the onset and progression of this metabolic disorder, and paved the way to the development of novel therapeutic strategies. Nonetheless, CC still remains a poorly defined syndrome, especially from the clinical standpoint. The main limitation probably lies in the fact that the precise role of individual CC mediators has remained undefined or controversial in several circumstances, thus complicating the process of establishing their clinical relevance. For instance, not all murine models of CC have shown involvement of the same set of signaling molecules during the disease development. For this reason, the idea of defining distinctive diagnostic and prognostic signatures of CC represents a considerable challenge for researchers and clinicians. In this scenario, the role of miRNAs as cell-to-cell mediators between the tumor cells and the surrounding microenvironment represents an interesting topic that deserves deeper investigation. Overall, these considerations highlight the urgent need to generate and validate new experimental models of CC, capable to accurately recapitulate molecular and histopathological aspects of human CC in a tumor type-dependent manner.

As systemic chronic inflammation and alternate metabolism are hallmarks of CC, the pharmacological targeting of individual underlying biomolecules has been considered as one possible effective approach to clinically manage this syndrome. Thus, several unimodal treatments have been developed in the attempt to properly manage this metabolic syndrome. Nonetheless, to date scarce or no effectiveness/safety has been reported for each of them. Given the complexity of CC, and considering the different patients' response to treatments through the many different clinical trials, it appears clear the impossibility to develop a general, universal therapy. On the contrary, it is crucial to define the cachectic patient's profile at both the molecular and clinical levels in order to reach a point where it is possible to provide the best customized nutritional support and treatment. Meanwhile, the emergence of further mediators of CC such as TLRs, PTHrP, and glucocorticoids, suggests the possibility to develop further safety pharmacological agents capable to prevent, or at least limit, the excessive triggering of catabolic processes and inflammation-related signaling.

Considering the multiplicity of metabolic and molecular aberrations, an important goal would be the development of appropriate multimodal approaches—intended as a balanced combination of pharmacological and non-pharmacological interventions—as recently suggested by several influential authors.

## Author Contributions

GM and FC wrote the manuscript. GM, PL, and FC contributed to the editing of the manuscript as needed.

### Conflict of Interest

The authors declare that the research was conducted in the absence of any commercial or financial relationships that could be construed as a potential conflict of interest.

## References

[1] Fearon K Arends J Baracos V. Understanding the mechanisms and treatment options in cancer cachexia. Nat Rev Clin Oncol. (2013) 10:90–9. 10.1038/nrclinonc.2012.209 23207794

[2] Schmidt SF Rohm M Herzig S Diaz MB. Cancer cachexia: more than skeletal muscle wasting. Trends Cancer. (2018) 4:849–60. 10.1016/j.trecan.2018.10.001 30470306

[3] Pin F Couch ME Bonetto A. Preservation of muscle mass as a strategy to reduce the toxic effects of cancer chemotherapy on body composition. Curr Opin Support Palliat Care. (2018) 12:420–6. 10.1097/SPC.0000000000000382 30124526 PMC6221433

[4] Evans WJ Morley JE Argilés J Bales C Baracos V Guttridge D. Cachexia: a new definition. Clin Nutr. (2008) 27:793–9. 10.1016/j.clnu.2008.06.013 18718696

[5] Smith KL Tisdale MJ. Increased protein degradation and decreased protein synthesis in skeletal muscle during cancer cachexia. Br J Cancer. (1993) 67:680–5. 10.1038/bjc.1993.126 8471425 PMC1968351

[6] Tisdale MJ. Cachexia in cancer patients. Nat Rev Cancer. (2002) 2:862–71. 10.1038/nrc927 12415256

[7] Penna F Baccino FM Costelli P. Coming back: autophagy in cachexia. Curr Opin Clin Nutr Metab Care. (2014) 17:241–6. 10.1097/MCO.0000000000000048 24535215

[8] Purcell SA Elliott SA Baracos VE Chu QSC Prado CM. Key determinants of energy expenditure in cancer and implications for clinical practice. Eur J Clin Nutr. (2016) 70:1230–8. 10.1038/ejcn.2016.96 27273068

[9] Vazeille C Jouinot A Durand J-P Neveux N Boudou-Rouquette P Huillard O. Relation between hypermetabolism, cachexia, and survival in cancer patients: a prospective study in 390 cancer patients before initiation of anticancer therapy. Am J Clin Nutr. (2017) 105:1139–47. 10.3945/ajcn.116.140434 28356274

[10] Kir S White JP Kleiner S Kazak L Cohen P Baracos VE. Tumour-derived PTH-related protein triggers adipose tissue browning and cancer cachexia. Nature. (2014) 513:100–4. 10.1038/nature13528 25043053 PMC4224962

[11] Petruzzelli M Schweiger M Schreiber R Campos-Olivas R Tsoli M Allen J. A switch from white to brown fat increases energy expenditure in cancer-associated cachexia. Cell Metab. (2014) 20:433–47. 10.1016/j.cmet.2014.06.011 25043816

[12] Zechner R Zimmermann R Eichmann TO Kohlwein SD Haemmerle G Lass A. FAT SIGNALS - lipases and lipolysis in lipid metabolism and signaling. Cell Metab. (2012) 15:279–91. 10.1016/j.cmet.2011.12.018 22405066 PMC3314979

[13] Arner P Langin D. Lipolysis in lipid turnover, cancer cachexia, and obesity-induced insulin resistance. Trends Endocrinol Metab. (2014) 25:255–62. 10.1016/j.tem.2014.03.002 24731595

[14] Fearon K Strasser F Anker SD Bosaeus I Bruera E Fainsinger RL. Definition and classification of cancer cachexia: an international consensus. Lancet Oncol. (2011) 12:489–95. 10.1016/S1470-2045(10)70218-7 21296615

[15] Anker MS Holcomb R Muscaritoli M von Haehling S Haverkamp W Jatoi A. Orphan disease status of cancer cachexia in the USA and in the European Union: a systematic review. J Cachexia Sarcopenia Muscle. (2019) 10:22–34. 10.1002/jcsm.12402 30920776 PMC6438416

[16] Arends J Bachmann P Baracos V Barthelemy N Bertz H Bozzetti F. ESPEN guidelines on nutrition in cancer patients. Clin Nutr. (2017) 36:11–48. 10.1016/j.clnu.2016.07.015 27637832

[17] Dewys WD Begg C Lavin PT Band PR Bennett JM Bertino JR. Prognostic effect of weight loss prior to chemotherapy in cancer patients. Eastern Cooperative Oncology Group. Am J Med. (1980) 69:491–7. 10.1016/S0149-2918(05)80001-3 7424938

[18] Hendifar A Yang D Lenz F Lurje G Pohl A Lenz C. Gender disparities in metastatic colorectal cancer survival. Clin Cancer Res. (2009) 15:6391–7. 10.1158/1078-0432.CCR-09-0877 19789331 PMC2779768

[19] Baracos VE Reiman T Mourtzakis M Gioulbasanis I Antoun S. Body composition in patients with non-small cell lung cancer: a contemporary view of cancer cachexia with the use of computed tomography image analysis. Am J Clin Nutr. (2010) 91:1133S−7S. 10.3945/ajcn.2010.28608C 20164322

[20] Baracos VE Martin L Korc M Guttridge DC Fearon KCH. Cancer-associated cachexia. Nat Rev Dis Primers. (2018) 4:17105. 10.1038/nrdp.2017.105 29345251

[21] Argilés JM Moore-Carrasco R Fuster G Busquets S López-Soriano FJ. Cancer cachexia: the molecular mechanisms. Int J Biochem Cell Biol. (2003) 35:405–9. 10.1016/S1357-2725(02)00251-0 12565701

[22] Prado CMM Bekaii-Saab T Doyle LA Shrestha S Ghosh S Baracos VE. Skeletal muscle anabolism is a side effect of therapy with the MEK inhibitor: selumetinib in patients with cholangiocarcinoma. Br J Cancer. (2012) 106:1583–6. 10.1038/bjc.2012.144 22510747 PMC3349178

[23] Prado CM Sawyer MB Ghosh S Lieffers JR Esfandiari N Antoun S. Central tenet of cancer cachexia therapy: do patients with advanced cancer have exploitable anabolic potential? Am J Clin Nutr. (2013) 98:1012–9. 10.3945/ajcn.113.060228 23966429

[24] Muscaritoli M Molfino A Lucia S Fanelli FR. Cachexia: a preventable comorbidity of cancer. A T.A.R.G.E.T. approach. Crit Rev Oncol Hematol. (2015) 94:251–9. 10.1016/j.critrevonc.2014.10.014 25468676

[25] Vanhoutte G van de Wiel M Wouters K Sels M Bartolomeeussen L De Keersmaecker S. Cachexia in cancer: what is in the definition? BMJ Open Gastroenterol. (2016) 3:e000097. 10.1136/bmjgast-2016-000097 27843571 PMC5093365

[26] Solheim TS Fayers PM Fladvad T Tan B Skorpen F Fearon K. Is there a genetic cause for cancer cachexia? - a clinical validation study in 1797 patients. Br J Cancer. (2011) 105:1244–51. 10.1038/bjc.2011.323 21934689 PMC3208484

[27] Johns N Stretch C Tan BHL Solheim TS Sørhaug S Stephens NA. New genetic signatures associated with cancer cachexia as defined by low skeletal muscle index and weight loss. J Cachexia Sarcopenia Muscle. (2017) 8:122–30. 10.1002/jcsm.12138 27897403 PMC5356227

[28] Borish LC Steinke JW. 2. Cytokines and chemokines. J Allergy Clin Immunol. (2003) 111:S460–75. 10.1067/mai.2003.108 12592293

[29] Seruga B Zhang H Bernstein LJ Tannock IF. Cytokines and their relationship to the symptoms and outcome of cancer. Nat Rev Cancer. (2008) 8:887–99. 10.1038/nrc2507 18846100

[30] Serhan CN Brain SD Buckley CD Gilroy DW Haslett C O'Neill LAJ. Resolution of inflammation: state of the art, definitions and terms. FASEB J. (2007) 21:325–32. 10.1096/fj.06-7227rev 17267386 PMC3119634

[31] Advani SM Advani PG VonVille HM Jafri SH. Pharmacological management of cachexia in adult cancer patients: a systematic review of clinical trials. BMC Cancer. (2018) 18:1174. 10.1186/s12885-018-5080-4 30482179 PMC6260745

[32] Beutler B Cerami A. Cachectin and tumour necrosis factor as two sides of the same biological coin. Nature. (1986) 320:584–8. 10.1038/320584a0 3010124

[33] Oliff A Defeo-Jones D Boyer M Martinez D Kiefer D Vuocolo G. Tumors secreting human TNF/cachectin induce cachexia in mice. Cell. (1987) 50:555–63. 10.1016/0092-8674(87)90028-6 3607879

[34] Aggarwal BB. Signalling pathways of the TNF superfamily: a double-edged sword. Nat Rev Immunol. (2003) 3:745–56. 10.1038/nri1184 12949498

[35] Guttridge DC Mayo MW Madrid LV Wang CY Baldwin AS Jr. NF-kappaB-induced loss of MyoD messenger RNA: possible role in muscle decay and cachexia. Science. (2000) 289:2363–6. 10.1126/science.289.5488.2363 11009425

[36] Ruan H Hacohen N Golub TR Van Parijs L Lodish HF. Tumor necrosis factor-alpha suppresses adipocyte-specific genes and activates expression of preadipocyte genes in 3T3-L1 adipocytes: nuclear factor-kappaB activation by TNF-alpha is obligatory. Diabetes. (2002) 51:1319–36. 10.2337/diabetes.51.5.1319 11978627

[37] Li YP Schwartz RJ Waddell ID Holloway BR Reid MB. Skeletal muscle myocytes undergo protein loss and reactive oxygen-mediated NF-kappaB activation in response to tumor necrosis factor alpha. FASEB J. (1998) 12:871–80. 10.1096/fasebj.12.10.871 9657527

[38] Li Y-P Reid MB. NF-κB mediates the protein loss induced by TNF-α in differentiated skeletal muscle myotubes. Am J Physiol Regul Integr Comp Physiol. (2000) 279:R1165–70. 10.1152/ajpregu.2000.279.4.R1165 11003979

[39] Acharyya S Ladner KJ Nelsen LL Damrauer J Reiser PJ Swoap S. Cancer cachexia is regulated by selective targeting of skeletal muscle gene products. J Clin Invest. (2004) 114:370–8. 10.1172/JCI200420174 15286803 PMC484974

[40] Dogra C Changotra H Wedhas N Qin X Wergedal JE Kumar A. TNF-related weak inducer of apoptosis. (TWEAK) is a potent skeletal muscle-wasting cytokine. FASEB J. (2007) 21:1857–69. 10.1096/fj.06-7537com 17314137 PMC4154373

[41] Mittal A Bhatnagar S Kumar A Lach-Trifilieff E Wauters S Li H. The TWEAK-Fn14 system is a critical regulator of denervation-induced skeletal muscle atrophy in mice. J Cell Biol. (2010) 188:833–49. 10.1083/jcb.200909117 20308426 PMC2845082

[42] Strassmann G Fong M Kenney JS Jacob CO. Evidence for the involvement of interleukin 6 in experimental cancer cachexia. J Clin Invest. (1992) 89:1681–4. 10.1172/JCI115767 1569207 PMC443047

[43] White JP Baynes JW Welle SL Kostek MC Matesic LE Sato S The regulation of skeletal muscle protein turnover during the progression of cancer cachexia in the ApcMin/mouse. PLoS ONE. (2011) 6:e24650 10.1371/journal.pone.0024650 21949739 PMC3176277

[44] Strassmann G Fong M Freter CE Windsor S D'Alessandro F Nordan RP. Suramin interferes with interleukin-6 receptor binding *in vitro* and inhibits colon-26-mediated experimental cancer cachexia *in vivo*. J Clin Invest. (1993) 92:2152–9. 10.1172/JCI116816 8227330 PMC288393

[45] Auernhammer CJ Melmed S. Leukemia-inhibitory factor-neuroimmune modulator of endocrine function. Endocr Rev. (2000) 21:313–45. 10.1210/er.21.3.313 10857556

[46] Hu W Feng Z Teresky AK Levine AJ. p53 regulates maternal reproduction through LIF. Nature. (2007) 450:721–4. 10.1038/nature05993 18046411

[47] Metcalf D Gearing DP. Fatal syndrome in mice engrafted with cells producing high levels of the leukemia inhibitory factor. Proc Natl Acad Sci USA. (1989) 86:5948–52. 10.1073/pnas.86.15.5948 2569739 PMC297748

[48] Akiyama Y Kajimura N Matsuzaki J Kikuchi Y Imai N Tanigawa M. *In vivo* effect of recombinant human leukemia inhibitory factor in primates. Jpn J Cancer Res. (1997) 88:578–83. 10.1111/j.1349-7006.1997.tb00421.x 9263535 PMC5921475

[49] Seto DN Kandarian SC Jackman RW. A key role for leukemia inhibitory factor in C26 cancer cachexia. J Biol Chem. (2015) 290:19976–86. 10.1074/jbc.M115.638411 26092726 PMC4528156

[50] Arora GK Gupta A Narayanan S Guo T Iyengar P Infante RE. Cachexia-associated adipose loss induced by tumor-secreted leukemia inhibitory factor is counterbalanced by decreased leptin. JCI Insight. (2018) 3:121221. 10.1172/jci.insight.121221 30046014 PMC6124433

[51] Baracos V Rodemann HP Dinarello CA Goldberg AL. Stimulation of muscle protein degradation and prostaglandin E2 release by leukocytic pyrogen. (interleukin-1). A mechanism for the increased degradation of muscle proteins during fever. N Engl J Med. (1983) 308:553–8. 10.1056/NEJM198303103081002 6402699

[52] Fong Y Moldawer LL Marano M Wei H Barber A Manogue K. Cachectin/TNF or IL-1 alpha induces cachexia with redistribution of body proteins. Am J Physiol Regul Integr Comp Physiol. (1989) 256:R659–65. 10.1152/ajpregu.1989.256.3.R659 2784290

[53] Gelin J Moldawer LL Lönnroth C Sherry B Chizzonite R Lundholm K. Role of endogenous tumor necrosis factor alpha and interleukin 1 for experimental tumor growth and the development of cancer cachexia. Cancer Res. (1991) 51:415–21. 1703040

[54] Braun TP Zhu X Szumowski M Scott GD Grossberg AJ Levasseur PR. Central nervous system inflammation induces muscle atrophy via activation of the hypothalamic-pituitary-adrenal axis. J Exp Med. (2011) 208:2449–63. 10.1084/jem.20111020 22084407 PMC3256966

[55] Robert F Mills JR Agenor A Wang D DiMarco S Cencic R. Targeting protein synthesis in a Myc/mTOR-driven model of anorexia-cachexia syndrome delays its onset and prolongs survival. Cancer Res. (2012) 72:747–56. 10.1158/0008-5472.CAN-11-2739 22158946

[56] Akdis M Burgler S Crameri R Eiwegger T Fujita H Gomez E. Interleukins, from 1 to 37, and interferon-γ: Receptors, functions, and roles in diseases. J Aller Clin Immunol. (2011) 127:701–21.e70. 10.1016/j.jaci.2010.11.050 21377040

[57] Ramani P Balkwill FR. Enhanced metastases of a mouse carcinoma after *in vitro* treatment with murine interferon gamma. Int J Cancer. (1987) 40:830–4. 10.1002/ijc.2910400621 3121523

[58] Ferrantini M Giovarelli M Modesti A Musiani P Modica A Venditti M. IFN-alpha 1 gene expression into a metastatic murine adenocarcinoma. (TS/A) results in CD8+ T cell-mediated tumor rejection and development of antitumor immunity. Comparative studies with IFN-gamma-producing TS/A cells. J Immunol. (1994) 153:4604–15. 7963533

[59] Billiau A Matthys P. Interferon-γ, more of a cachectin than tumor necrosis factor. Cytokine. (1992) 4:259–63. 10.1016/1043-4666(92)90065-Y 1515549

[60] Matthys P Dijkmans R Proost P Van Damme J Heremans H Sobis H. Severe cachexia in mice inoculated with interferon-gamma-producing tumor cells. Int J Cancer. (1991) 49:77–82. 10.1002/ijc.2910490115 1908442

[61] Matthys P Heremans H Opdenakker G Billiau A. Anti-interferon-γ antibody treatment, growth of Lewis lung tumours in mice and tumour-associated cachexia. Eur J Cancer Clin Oncol. (1991) 27:182–7. 10.1016/0277-5379(91)90483-T 1827286

[62] Iorio MV Croce CM. Causes and consequences of MicroRNA dysregulation. Cancer J. (2012) 18:215–22. 10.1097/PPO.0b013e318250c001 22647357 PMC3528102

[63] Nana-Sinkam SP Croce CM. Clinical applications for microRNAs in cancer. Clin Pharmacol Ther. (2013) 93:98–104. 10.1038/clpt.2012.192 23212103

[64] O'Brien J Hayder H Zayed Y Peng C. Overview of MicroRNA biogenesis, mechanisms of actions, and circulation. Front Endocrinol. (2018) 9:402. 10.3389/fendo.2018.00402 30123182 PMC6085463

[65] Zhou W Fong MY Min Y Somlo G Liu L Palomares MR. Cancer-secreted miR-105 destroys vascular endothelial barriers to promote metastasis. Cancer Cell. (2014) 25:501–15. 10.1016/j.ccr.2014.03.007 24735924 PMC4016197

[66] He WA Calore F Londhe P Canella A Guttridge DC Croce CM. Microvesicles containing miRNAs promote muscle cell death in cancer cachexia via TLR7. Proc Natl Acad Sci USA. (2014) 111:4525–9. 10.1073/pnas.1402714111 24616506 PMC3970508

[67] Okugawa Y Toiyama Y Hur K Yamamoto A Yin C Ide S. Circulating miR-203 derived from metastatic tissues promotes myopenia in colorectal cancer patients. J Cachexia Sarcopenia Muscle. (2019) 10:536–48. 10.1002/jcsm.12403 31091026 PMC6596405

[68] Soares RJ Cagnin S Chemello F Silvestrin M Musaro A De Pitta C. Involvement of microRNAs in the regulation of muscle wasting during catabolic conditions. J Biol Chem. (2014) 289:21909–25. 10.1074/jbc.M114.561845 24891504 PMC4139209

[69] Wu Q Sun S Li Z Yang Q Li B Zhu S. Tumour-originated exosomal miR-155 triggers cancer-associated cachexia to promote tumour progression. Mol Cancer. (2018) 17:155. 10.1186/s12943-018-0899-5 30359265 PMC6201501

[70] Gay NJ Symmons MF Gangloff M Bryant CE. Assembly and localization of Toll-like receptor signalling complexes. Nat Rev Immunol. (2014) 14:546–58. 10.1038/nri3713 25060580

[71] Bohnert KR Goli P Roy A Sharma AK Xiong G Gallot YS. The toll-like receptor/MyD88/XBP1 signaling axis mediates skeletal muscle wasting during cancer cachexia. Mol Cell Biol. (2019) 39: e00184-19. 10.1128/MCB.00184-19 31138662 PMC6639248

[72] Doyle A Zhang G Abdel Fattah EA Eissa NT Li Y-P. Toll-like receptor 4 mediates lipopolysaccharide-induced muscle catabolism via coordinate activation of ubiquitin-proteasome and autophagy-lysosome pathways. FASEB J. (2011) 25:99–110. 10.1096/fj.10-164152 20826541 PMC3005430

[73] Zhang G Liu Z Ding H Zhou Y Doan HA Sin KWT. Tumor induces muscle wasting in mice through releasing extracellular Hsp70 and Hsp90. Nat Commun. (2017) 8:589. 10.1038/s41467-017-00726-x 28928431 PMC5605540

[74] Henriques F Lopes MA Franco FO Knobl P Santos KB Bueno LL. Toll-like receptor-4 disruption suppresses adipose tissue remodeling and increases survival in cancer cachexia syndrome. Sci Rep. (2018) 8:18024. 10.1038/s41598-018-36626-3 30575787 PMC6303407

[75] Calore F Londhe P Fadda P Nigita G Casadei L Marceca GP. The TLR7/8/9 antagonist IMO-8503 inhibits cancer-induced cachexia. Cancer Res. (2018) 78:6680–90. 10.1158/0008-5472.CAN-17-3878 30209066 PMC6541227

[76] Terawaki K Kashiwase Y Uzu M Nonaka M Sawada Y Miyano K. Leukemia inhibitory factor via the Toll-like receptor 5 signaling pathway involves aggravation of cachexia induced by human gastric cancer-derived 85As2 cells in rats. Oncotarget. (2018) 9:34748–64. 10.18632/oncotarget.26190 30410674 PMC6205166

[77] Morvan F Rondeau J-M Zou C Minetti G Scheufler C Scharenberg M. Blockade of activin type II receptors with a dual anti-ActRIIA/IIB antibody is critical to promote maximal skeletal muscle hypertrophy. Proc Natl Acad Sci USA. (2017) 114:12448–53. 10.1073/pnas.1707925114 29109273 PMC5703284

[78] Joulia-Ekaza D Cabello G. The myostatin gene: physiology and pharmacological relevance. Curr Opin Pharmacol. (2007) 7:310–5. 10.1016/j.coph.2006.11.011 17374508

[79] Zimmers TA Davies MV Koniaris LG Haynes P Esquela AF Tomkinson KN. Induction of cachexia in mice by systemically administered myostatin. Science. (2002) 296:1486–8. 10.1126/science.1069525 12029139

[80] Bogdanovich S Krag TOB Barton ER Morris LD Whittemore L-A Ahima RS. Functional improvement of dystrophic muscle by myostatin blockade. Nature. (2002) 420:418–21. 10.1038/nature01154 12459784

[81] Welle S Bhatt K Pinkert CA Tawil R Thornton CA. Muscle growth after postdevelopmental myostatin gene knockout. Am J Physiol Endocrinol Metab. (2007) 292:E985–91. 10.1152/ajpendo.00531.2006 17148752

[82] Costelli P Muscaritoli M Bonetto A Penna F Reffo P Bossola M. Muscle myostatin signalling is enhanced in experimental cancer cachexia. Eur J Clin Invest. (2008) 38:531–8. 10.1111/j.1365-2362.2008.01970.x 18578694

[83] Liu C-M Yang Z Liu C-W Wang R Tien P Dale R. Myostatin antisense RNA-mediated muscle growth in normal and cancer cachexia mice. Gene Ther. (2008) 15:155–60. 10.1038/sj.gt.3303016 18033313

[84] Xia Y Schneyer AL. The biology of activin: recent advances in structure, regulation and function. J Endocrinol. (2009) 202:1–12. 10.1677/JOE-08-0549 19273500 PMC2704481

[85] Hedger MP de Kretser DM. The activins and their binding protein, follistatin-Diagnostic and therapeutic targets in inflammatory disease and fibrosis. Cytok Growth Factor Rev. (2013) 24:285–95. 10.1016/j.cytogfr.2013.03.003 23541927

[86] Leto G Incorvaia L Badalamenti G Tumminello FM Gebbia N Flandina C Activin A circulating levels in patients with bone metastasis from breast or prostate cancer. Clin Exp Metast. (2006) 23:117–22. 10.1007/s10585-006-9010-5 16841234

[87] Zhou X Wang JL Lu J Song Y Kwak KS Jiao Q. Reversal of cancer cachexia and muscle wasting by ActRIIB antagonism leads to prolonged survival. Cell. (2010) 142:531–43. 10.1016/j.cell.2010.07.011 20723755

[88] Chen JL Walton KL Winbanks CE Murphy KT Thomson RE Makanji Y. Elevated expression of activins promotes muscle wasting and cachexia. FASEB J. (2014) 28:1711–23. 10.1096/fj.13-245894 24378873

[89] Busquets S Toledo M Orpí M Massa D Porta M Capdevila E. Myostatin blockage using actRIIB antagonism in mice bearing the Lewis lung carcinoma results in the improvement of muscle wasting and physical performance. J Cachexia Sarcopenia Muscle. (2012) 3:37–43. 10.1007/s13539-011-0049-z 22450815 PMC3302990

[90] Kir S Komaba H Garcia AP Economopoulos KP Liu W Lanske B. PTH/PTHrP receptor mediates cachexia in models of kidney failure and cancer. Cell Metab. (2016) 23:315–23. 10.1016/j.cmet.2015.11.003 26669699 PMC4749423

[91] Vilardaga J-P Romero G Friedman PA Gardella TJ. Molecular basis of parathyroid hormone receptor signaling and trafficking: a family B GPCR paradigm. Cell Mol Life Sci. (2011) 68:1–13. 10.1007/s00018-010-0465-9 20703892 PMC3568769

[92] Bao Y Bing C Hunter L Jenkins JR Wabitsch M Trayhurn P. Zinc-alpha2-glycoprotein, a lipid mobilizing factor, is expressed and secreted by human. (SGBS) adipocytes. FEBS Lett. (2005) 579:41–7. 10.1016/j.febslet.2004.11.042 15620688

[93] Bing C Bao Y Jenkins J Sanders P Manieri M Cinti S Zinc- 2-glycoprotein, a lipid mobilizing factor, is expressed in adipocytes and is up-regulated in mice with cancer cachexia. Proc Natl Acad Sci USA. (2004) 101:2500–5. 10.1073/pnas.0308647100 14983038 PMC356979

[94] Rydén M Agustsson T Andersson J Bolinder J Toft E Arner P. Adipose zinc-α2-glycoprotein is a catabolic marker in cancer and noncancerous states. J Intern Med. (2012) 271:414–20. 10.1111/j.1365-2796.2011.02441.x 21883534

[95] Russell ST Zimmerman TP Domin BA Tisdale MJ. Induction of lipolysis *in vitro* and loss of body fat *in vivo* by zinc-α2-glycoprotein. Biochim Biophys Acta. Mol Cell Biol Lipids. (2004) 1636:59–68. 10.1016/j.bbalip.2003.12.004 14984739

[96] Russell ST Tisdale MJ. Effect of eicosapentaenoic acid. (EPA) on expression of a lipid mobilizing factor in adipose tissue in cancer cachexia. Prostaglandins Leukot Essent Fatty Acids. (2005) 72:409–14. 10.1016/j.plefa.2005.03.002 15899583

[97] Rolli V Radosavljevic M Astier V Macquin C Castan-Laurell I Visentin V Lipolysis is altered in MHC class I zinc-α2-glycoprotein deficient mice. FEBS Lett. (2007) 581:394–400. 10.1016/j.febslet.2006.12.047 17234189

[98] Argilés JM López-Soriano FJ. The ubiquitin-dependent proteolytic pathway in skeletal muscle: its role in pathological states. Trends Pharmacol Sci. (1996) 17:223–6. 10.1016/0165-6147(96)10021-3 8763200

[99] Glass DJ. Signaling pathways perturbing muscle mass. Curr Opin Clin Nutr Metab Care. (2010) 13:225–9. 10.1097/MCO.0b013e32833862df 20397318

[100] Zhang G Jin B Li Y-P. C/EBPβ mediates tumour-induced ubiquitin ligase atrogin1/MAFbx upregulation and muscle wasting. EMBO J. (2011) 30:4323–35. 10.1038/emboj.2011.292 21847090 PMC3199382

[101] He WA Berardi E Cardillo VM Acharyya S Aulino P Thomas-Ahner J. NF-κB-mediated Pax7 dysregulation in the muscle microenvironment promotes cancer cachexia. J Clin Invest. (2013) 123:4821–35. 10.1172/JCI68523 24084740 PMC3809785

[102] Zimmers TA Fishel ML Bonetto A. STAT3 in the systemic inflammation of cancer cachexia. Semin Cell Dev Biol. (2016) 54:28–41. 10.1016/j.semcdb.2016.02.009 26860754 PMC4867234

[103] Ma JF Sanchez BJ Hall DT Tremblay AK Di Marco S Gallouzi I STAT 3 promotes IFN γ/ TNF α-induced muscle wasting in an NF -κB-dependent and IL−6-independent manner. EMBO Mol Med. (2017) 9:622–37. 10.15252/emmm.201607052 28264935 PMC5412921

[104] Stitt TN Drujan D Clarke BA Panaro F Timofeyva Y Kline WO. The IGF-1/PI3K/Akt pathway prevents expression of muscle atrophy-induced ubiquitin ligases by inhibiting FOXO transcription factors. Mol Cell. (2004) 14:395–403. 10.1016/S1097-2765(04)00211-4 15125842

[105] Sandri M Sandri C Gilbert A Skurk C Calabria E Picard A. Foxo transcription factors induce the atrophy-related ubiquitin ligase atrogin-1 and cause skeletal muscle atrophy. Cell. (2004) 117:399–412. 10.1016/S0092-8674(04)00400-3 15109499 PMC3619734

[106] Ben-Sahra I Manning BD. mTORC1 signaling and the metabolic control of cell growth. Curr Opin Cell Biol. (2017) 45:72–82. 10.1016/j.ceb.2017.02.012 28411448 PMC5545101

[107] Song Y-H Li Y Du J Mitch WE Rosenthal N Delafontaine P. Muscle-specific expression of IGF-1 blocks angiotensin II-induced skeletal muscle wasting. J Clin Investig. (2005) 115:451–8. 10.1172/JCI22324 15650772 PMC544037

[108] Milan G Romanello V Pescatore F Armani A Paik J-H Frasson L. Regulation of autophagy and the ubiquitin-proteasome system by the FoxO transcriptional network during muscle atrophy. Nat Commun. (2015) 6:6670. 10.1038/ncomms7670 25858807 PMC4403316

[109] Sandri M Lin J Handschin C Yang W Arany ZP Lecker SH. PGC-1alpha protects skeletal muscle from atrophy by suppressing FoxO3 action and atrophy-specific gene transcription. Proc Natl Acad Sci USA. (2006) 103:16260–5. 10.1073/pnas.0607795103 17053067 PMC1637570

[110] Patra MC Shah M Choi S. Toll-like receptor-induced cytokines as immunotherapeutic targets in cancers and autoimmune diseases. Semin Cancer Biol. (in press). 10.1016/j.semcancer.2019.05.002 31054927

[111] Wang EL Qian ZR Nakasono M Tanahashi T Yoshimoto K Bando Y. High expression of Toll-like receptor 4/myeloid differentiation factor 88 signals correlates with poor prognosis in colorectal cancer. Br J Cancer. (2010) 102:908–15. 10.1038/sj.bjc.6605558 20145615 PMC2833250

[112] Sun Q Liu Q Zheng Y Cao X Rapamycin suppresses TLR4-triggered IL-6 and PGE2 production of colon cancer cells by inhibiting TLR4 expression and NF-κB activation. Mol Immunol. (2008) 45:2929–36. 10.1016/j.molimm.2008.01.025 18343502

[113] Kundu SD Lee C Billips BK Habermacher GM Zhang Q Liu V. The toll-like receptor pathway: a novel mechanism of infection-induced carcinogenesis of prostate epithelial cells. Prostate. (2008) 68:223–9. 10.1002/pros.20710 18092352

[114] Killeen SD Wang JH Andrews EJ Redmond HP. Bacterial endotoxin enhances colorectal cancer cell adhesion and invasion through TLR-4 and NF-κB-dependent activation of the urokinase plasminogen activator system. Br J Cancer. (2009) 100:1589–602. 10.1038/sj.bjc.6604942 19436306 PMC2696751

[115] Argilés JM Orpí M Busquets S López-Soriano FJ. Myostatin: more than just a regulator of muscle mass. Drug Discov Today. (2012) 17:702–9. 10.1016/j.drudis.2012.02.001 22342983

[116] Kemaladewi DU de Gorter DJJ Aartsma-Rus A van Ommen G-J ten Dijke P 't PA. Cell-type specific regulation of myostatin signaling. FASEB J. (2012) 26:1462–72. 10.1096/fj.11-191189 22202673

[117] Penna F Costamagna D Pin F Camperi A Fanzani A Chiarpotto EM. Autophagic degradation contributes to muscle wasting in cancer cachexia. Am J Pathol. (2013) 182:1367–78. 10.1016/j.ajpath.2012.12.023 23395093

[118] Tardif N Klaude M Lundell L Thorell A Rooyackers O. Autophagic-lysosomal pathway is the main proteolytic system modified in the skeletal muscle of esophageal cancer patients. Am J Clin Nutr. (2013) 98:1485–92. 10.3945/ajcn.113.063859 24108784

[119] Aversa Z Pin F Lucia S Penna F Verzaro R Fazi M. Autophagy is induced in the skeletal muscle of cachectic cancer patients. Sci Rep. (2016) 6:30340. 10.1038/srep30340 27459917 PMC4962093

[120] Pigna E Berardi E Aulino P Rizzuto E Zampieri S Carraro U. Aerobic exercise and pharmacological treatments counteract cachexia by modulating autophagy in colon cancer. Sci Rep. (2016) 6:26991. 10.1038/srep26991 27244599 PMC4886631

[121] Liu Z Sin KWT Ding H Doan HA Gao S Miao H. p38β MAPK mediates ULK1-dependent induction of autophagy in skeletal muscle of tumor-bearing mice. Cell Stress Chaperones. (2018) 2:311–24. 10.15698/cst2018.11.163 31225455 PMC6551802

[122] Coppack SW. Pro-inflammatory cytokines and adipose tissue. Proc Nutr Soc. (2001) 60:349–56. 10.1079/PNS2001110 11681809

[123] Mead JR Irvine SA Ramji DP. Lipoprotein lipase: structure, function, regulation, and role in disease. J Mol Med. (2002) 80:753–69. 10.1007/s00109-002-0384-9 12483461

[124] Thompson MP Cooper ST Parry BR Tuckey JA. Increased expression of the mRNA for hormone-sensitive lipase in adipose tissue of cancer patients. Biochim Biophys Acta. (1993) 1180:236–42. 10.1016/0925-4439(93)90044-2 8422428

[125] Beck SA Tisdale MJ. Effect of cancer cachexia on triacylglycerol/fatty acid substrate cycling in white adipose tissue. Lipids. (2004) 39:1187–9. 10.1007/s11745-004-1346-8 15736914

[126] Zhang HH Halbleib M Ahmad F Manganiello VC Greenberg AS. Tumor necrosis factor- stimulates lipolysis in differentiated human adipocytes through activation of extracellular signal-related kinase and elevation of intracellular cAMP. Diabetes. (2002) 51:2929–35. 10.2337/diabetes.51.10.2929 12351429

[127] Hirai K Hussey HJ Barber MD Price SA Tisdale MJ. Biological evaluation of a lipid-mobilizing factor isolated from the urine of cancer patients. Cancer Res. (1998) 58:2359–65. 9622075

[128] Islam-Ali B Khan S Price SA Tisdale MJ. Modulation of adipocyte G-protein expression in cancer cachexia by a lipid-mobilizing factor. (LMF). Br J Cancer. (2001) 85:758–63. 10.1054/bjoc.2001.1992 11531264 PMC2364135

[129] Tsoli M Schweiger M Vanniasinghe AS Painter A Zechner R Clarke S. Depletion of white adipose tissue in cancer cachexia syndrome is associated with inflammatory signaling and disrupted circadian regulation. PLoS ONE. (2014) 9:e92966. 10.1371/journal.pone.0092966 24667661 PMC3965507

[130] Lowell BB Spiegelman BM. Towards a molecular understanding of adaptive thermogenesis. Nature. (2000) 404:652–60. 10.1038/35007527 10766252

[131] Bing C Brown M King P Collins P Tisdale MJ Williams G. Increased gene expression of brown fat uncoupling protein. (UCP)1 and skeletal muscle UCP2 and UCP3 in MAC16-induced cancer cachexia. Cancer Res. (2000) 60:2405–10. 10.1042/cs098001Pa 10811117

[132] Collins P Bing C McCulloch P Williams G. Muscle UCP-3 mRNA levels are elevated in weight loss associated with gastrointestinal adenocarcinoma in humans. Br J Cancer. (2002) 86:372–5. 10.1038/sj.bjc.6600074 11875702 PMC2375209

[133] Kliewer KL Ke J-Y Tian M Cole RM Andridge RR Belury MA. Adipose tissue lipolysis and energy metabolism in early cancer cachexia in mice. Cancer Biol Ther. (2015) 16:886–97. 10.4161/15384047.2014.987075 25457061 PMC4622729

[134] Petrovic N Walden TB Shabalina IG Timmons JA Cannon B Nedergaard J. Chronic peroxisome proliferator-activated receptor γ. (PPARγ) activation of epididymally derived white adipocyte cultures reveals a population of thermogenically competent, UCP1-containing adipocytes molecularly distinct from classic brown adipocytes. J Biol Chem. (2010) 285:7153–64. 10.1074/jbc.M109.053942 20028987 PMC2844165

[135] Villarroya F Cereijo R Villarroya J Giralt M. Brown adipose tissue as a secretory organ. Nat Rev Endocrinol. (2017) 13:26–35. 10.1038/nrendo.2016.136 27616452

[136] Kimura S Yoshioka K. Parathyroid hormone and parathyroid hormone type-1 receptor accelerate myocyte differentiation. Sci Rep. (2014) 4:5066. 10.1038/srep05066 24919035 PMC4052750

[137] Tsoli M Moore M Burg D Painter A Taylor R Lockie SH. Activation of thermogenesis in brown adipose tissue and dysregulated lipid metabolism associated with cancer cachexia in mice. Cancer Res. (2012) 72:4372–82. 10.1158/0008-5472.CAN-11-3536 22719069

[138] Cao W Daniel KW Robidoux J Puigserver P Medvedev AV Bai X. p38 mitogen-activated protein kinase is the central regulator of cyclic AMP-dependent transcription of the brown fat uncoupling protein 1 gene. Mol Cell Biol. (2004) 24:3057–67. 10.1128/MCB.24.7.3057-3067.2004 15024092 PMC371122

[139] Li G Klein RL Matheny M King MA Meyer EM Scarpace PJ. Induction of uncoupling protein 1 by central interleukin-6 gene delivery is dependent on sympathetic innervation of brown adipose tissue and underlies one mechanism of body weight reduction in rats. Neuroscience. (2002) 115:879–89. 10.1016/S0306-4522(02)00447-5 12435426

[140] Wallenius K Wallenius V Sunter D Dickson SL Jansson J-O. Intracerebroventricular interleukin-6 treatment decreases body fat in rats. Biochem Biophys Res Commun. (2002) 293:560–5. 10.1016/S0006-291X(02)00230-9 12054638

[141] Nguyen KD Qiu Y Cui X Goh YPS Mwangi J David T. Alternatively activated macrophages produce catecholamines to sustain adaptive thermogenesis. Nature. (2011) 480:104–8. 10.1038/nature10653 22101429 PMC3371761

[142] Mauer J Chaurasia B Goldau J Vogt MC Ruud J Nguyen KD. Signaling by IL-6 promotes alternative activation of macrophages to limit endotoxemia and obesity-associated resistance to insulin. Nat Immunol. (2014) 15:423–30. 10.1038/ni.2865 24681566 PMC4161471

[143] Baracos VE. Bridging the gap: are animal models consistent with clinical cancer cachexia? Nat Rev Clin Oncol. (2018) 15:197–8. 10.1038/nrclinonc.2018.14 29405197

[144] Talbert EE Cuitiño MC Ladner KJ Rajasekerea PV Siebert M Shakya R. Modeling human cancer-induced cachexia. Cell Rep. (2019) 28:1612–22.e4. 10.1016/j.celrep.2019.07.016 31390573 PMC6733019

[145] Cernackova A Mikova L Horvathova L Tillinger A Mravec B Cachexia induced by Yoshida ascites hepatoma in Wistar rats is not associated with inflammatory response in the spleen or brain. J Neuroimmunol. (2019) 337:577068 10.1016/j.jneuroim.2019.577068 31606594

[146] Vooijs M Jonkers J Berns A. A highly efficient ligand-regulated Cre recombinase mouse line shows that LoxP recombination is position dependent. EMBO Rep. (2001) 2:292–7. 10.1093/embo-reports/kve064 11306549 PMC1083861

[147] Branda CS Dymecki SM. Talking about a revolution. Dev Cell. (2004) 6:7–28. 10.1016/S1534-5807(03)00399-X 14723844

[148] Kristianto J Johnson MG Zastrow RK Radcliff AB Blank RD. Spontaneous recombinase activity of Cre-ERT2 *in vivo*. Trans Res. (2017) 26:411–7. 10.1007/s11248-017-0018-1 28409408 PMC9474299

[149] Hingorani SR Wang L Multani AS Combs C Deramaudt TB Hruban RH. Trp53R172H and KrasG12D cooperate to promote chromosomal instability and widely metastatic pancreatic ductal adenocarcinoma in mice. Cancer Cell. (2005) 7:469–83. 10.1016/j.ccr.2005.04.023 15894267

[150] Michaelis KA Zhu X Burfeind KG Krasnow SM Levasseur PR Morgan TK. Establishment and characterization of a novel murine model of pancreatic cancer cachexia. J Cachexia Sarcopenia Muscle. (2017) 8:824–38. 10.1002/jcsm.12225 28730707 PMC5659050

[151] Parajuli P Kumar S Loumaye A Singh P Eragamreddy S Nguyen TL. Twist1 activation in muscle progenitor cells causes muscle loss akin to cancer cachexia. Dev Cell. (2018) 45:712–25.e6. 10.1016/j.devcel.2018.05.026 29920276 PMC6054474

[152] Goncalves MD Hwang S-K Pauli C Murphy CJ Cheng Z Hopkins BD. Fenofibrate prevents skeletal muscle loss in mice with lung cancer. Proc Natl Acad Sci USA. (2018) 115:E743–52. 10.1073/pnas.1714703115 29311302 PMC5789923

[153] Maitra R Thavornwatanayong T Venkatesh MK Chandy C Vachss D Augustine T. Development and characterization of a genetic mouse model of KRAS mutated colorectal cancer. Int J Mol Sci. (2019) 20:E5677. 10.3390/ijms20225677 31766149 PMC6888417

[154] Delitto D Pham K Vlada AC Sarosi GA Thomas RM Behrns KE. Patient-derived xenograft models for pancreatic adenocarcinoma demonstrate retention of tumor morphology through incorporation of murine stromal elements. Am J Pathol. (2015) 185:1297–303. 10.1016/j.ajpath.2015.01.016 25770474 PMC4419203

[155] Pham K Delitto D Knowlton AE Hartlage ER Madhavan R Gonzalo DH. Isolation of pancreatic cancer cells from a patient-derived xenograft model allows for practical expansion and preserved heterogeneity in culture. Am J Pathol. (2016) 186:1537–46. 10.1016/j.ajpath.2016.02.009 27102771 PMC4901138

[156] Go KL Delitto D Judge SM Gerber MH George TJ Jr Behrns KE. Orthotopic patient-derived pancreatic cancer xenografts engraft into the pancreatic parenchyma, metastasize, and induce muscle wasting to recapitulate the human disease. Pancreas. (2017) 46:813–9. 10.1097/MPA.0000000000000843 28609371 PMC7094873

[157] Michaelis KA Norgard MA Zhu X Levasseur PR Sivagnanam S Liudahl SM. Publisher correction: the TLR7/8 agonist R848 remodels tumor and host responses to promote survival in pancreatic cancer. Nat Commun. (2019) 10:5257. 10.1038/s41467-019-13151-z 31729370 PMC6858433

[158] Sadeghi M Keshavarz-Fathi M Baracos V Arends J Mahmoudi M Rezaei N. Cancer cachexia: diagnosis, assessment, and treatment. Crit Rev Oncol Hematol. (2018) 127:91–104. 10.1016/j.critrevonc.2018.05.006 29891116

[159] Aversa Z Costelli P Muscaritoli M. Cancer-induced muscle wasting: latest findings in prevention and treatment. Ther Adv Med Oncol. (2017) 9:369–82. 10.1177/1758834017698643 28529552 PMC5424865

[160] Anderson LJ Albrecht ED Garcia JM. Erratum to: update on management of cancer-related cachexia. Curr Oncol Rep. (2017) 19:22. 10.1007/s11912-017-0595-4 28293870

[161] Jatoi A Dakhil SR Nguyen PL Sloan JA Kugler JW Rowland KM Jr. A placebo-controlled double blind trial of etanercept for the cancer anorexia/weight loss syndrome: results from N00C1 from the North Central Cancer Treatment Group. Cancer. (2007) 110:1396–403. 10.1002/cncr.22944 17674351

[162] Gordon JN Trebble TM Ellis RD Duncan HD Johns T Goggin PM. Thalidomide in the treatment of cancer cachexia: a randomised placebo controlled trial. Gut. (2005) 54:540–5. 10.1136/gut.2004.047563 15753541 PMC1774430

[163] Mantovani G. Randomised phase III clinical trial of 5 different arms of treatment on 332 patients with cancer cachexia. Eur Rev Med Pharmacol Sci. (2010) 14:292–301. 20496538

[164] Yennurajalingam S Willey JS Palmer JL Allo J Del Fabbro E Cohen EN. The role of thalidomide and placebo for the treatment of cancer-related anorexia-cachexia symptoms: results of a double-blind placebo-controlled randomized study. J Palliat Med. (2012) 15:1059–64. 10.1089/jpm.2012.0146 22880820 PMC3438834

[165] Mehrzad V Afshar R Akbari M. Pentoxifylline treatment in patients with cancer cachexia: a double-blind, randomized, placebo-controlled clinical trial. Adv Biomed Res. (2016) 5:60. 10.4103/2277-9175.179182 27135029 PMC4832883

[166] Goldberg RM Loprinzi CL Mailliard JA O'Fallon JR Krook JE Ghosh C. Pentoxifylline for treatment of cancer anorexia and cachexia? A randomized, double-blind, placebo-controlled trial. J Clin Oncol. (1995) 13:2856–9. 10.1200/JCO.1995.13.11.2856 7595749

[167] Jatoi A Ritter HL Dueck A Nguyen PL Nikcevich DA Luyun RF. A placebo-controlled, double-blind trial of infliximab for cancer-associated weight loss in elderly and/or poor performance non-small cell lung cancer patients. (N01C9). Lung Cancer. (2010) 68:234–9. 10.1016/j.lungcan.2009.06.020 19665818 PMC5951722

[168] Clarke SJ Smith JT Gebbie C Sweeney C Olszewski N A phase I, pharmacokinetic. (PK), and preliminary efficacy assessment of ALD518, a humanized anti-IL-6 antibody, in patients with advanced cancer. J Clin Oncol. (2009) 27:3025 10.1200/jco.2009.27.15_suppl.3025

[169] Rigas JR Schuster M Orlov SV Milovanovic B Prabhash K Smith JT Efect of ALD518, a humanized anti-IL-6 antibody, on lean body mass loss and symptoms in patients with advanced non-small cell lung cancer. (NSCLC): results of a phase II randomized, double-blind safety and efficacy trial. J Clin Oncol. (2010) 28:7622 10.1200/jco.2010.28.15_suppl.7622

[170] Bekaii-Saab T Phelps MA Li X Saji M Goff L Kauh JSW. Multi-institutional phase II study of selumetinib in patients with metastatic biliary cancers. J Clin Oncol. (2011) 29:2357–63. 10.1200/JCO.2010.33.9473 21519026 PMC3107751

[171] Hong DS Hui D Bruera E Janku F Naing A Falchook GS. MABp1, a first-in-class true human antibody targeting interleukin-1α in refractory cancers: an open-label, phase 1 dose-escalation and expansion study. Lancet Oncol. (2014) 15:656–66. 10.1016/S1470-2045(14)70155-X 24746841

[172] Hickish T Andre T Wyrwicz L Saunders M Sarosiek T Kocsis J. MABp1 as a novel antibody treatment for advanced colorectal cancer: a randomised, double-blind, placebo-controlled, phase 3 study. Lancet Oncol. (2017) 18:192–201. 10.1016/S1470-2045(17)30006-2 28094194

[173] Lai V George J Richey L Kim HJ Cannon T Shores C. Results of a pilot study of the effects of celecoxib on cancer cachexia in patients with cancer of the head, neck, and gastrointestinal tract. Head Neck. (2008) 30:67–74. 10.1002/hed.20662 17615567

[174] Mantovani G Macciò A Madeddu C Serpe R Antoni G Massa E. Phase II nonrandomized study of the efficacy and safety of COX-2 inhibitor celecoxib on patients with cancer cachexia. J Mol Med. (2010) 88:85–92. 10.1007/s00109-009-0547-z 19802504

[175] Jameson GS Von Hoff DD Weiss GJ Richards DA Smith DA Becerra C Safety of the antimyostatin monoclonal antibody LY2495655 in healthy subjects and patients with advanced cancer. J Clin Oncol. (2012) 30:2516 10.1200/jco.2012.30.15_suppl.251622614993

[176] Golan T Geva R Richards D Madhusudan S Lin BK Wang HT. LY2495655, an antimyostatin antibody, in pancreatic cancer: a randomized, phase 2 trial. J Cachexia Sarcopenia Muscle. (2018) 9:871–9. 10.1002/jcsm.12331 30051975 PMC6204586

[177] Padhi D Higano CS Shore ND Sieber P Rasmussen E Smith MR. Pharmacological inhibition of myostatin and changes in lean body mass and lower extremity muscle size in patients receiving androgen deprivation therapy for prostate cancer. J Clin Endocrinol Metab. (2014) 99:E1967–75. 10.1210/jc.2014-1271 24971661

[178] Ruiz Garcia V López-Briz E Carbonell Sanchis R Gonzalvez Perales JL Bort-Marti S. Megestrol acetate for treatment of anorexia-cachexia syndrome. Cochrane Database Syst Rev. (2013) CD004310. 10.1002/14651858.CD004310.pub3 23543530 PMC6418472

[179] Madeddu C Macciò A Panzone F Tanca FM Mantovani G. Medroxyprogesterone acetate in the management of cancer cachexia. Expert Opin Pharmacother. (2009) 10:1359–66. 10.1517/14656560902960162 19445562

[180] Hiura Y Takiguchi S Yamamoto K Takahashi T Kurokawa Y Yamasaki M. Effects of ghrelin administration during chemotherapy with advanced esophageal cancer patients: a prospective, randomized, placebo-controlled phase 2 study. Cancer. (2012) 118:4785–94. 10.1002/cncr.27430 22282373

[181] Temel JS Abernethy AP Currow DC Friend J Duus EM Yan Y. Anamorelin in patients with non-small-cell lung cancer and cachexia. (ROMANA 1 and ROMANA 2): results from two randomised, double-blind, phase 3 trials. Lancet Oncol. (2016) 17:519–31. 10.1016/S1470-2045(15)00558-6 26906526

[182] Currow D Temel JS Abernethy A Milanowski J Friend J Fearon KC. ROMANA 3: a phase 3 safety extension study of anamorelin in advanced non-small-cell lung cancer. (NSCLC) patients with cachexia. Ann Oncol. (2017) 28:1949–56. 10.1093/annonc/mdx192 28472437 PMC5834076

[183] Cannabis-In-Cachexia-Study-Group Strasser F Luftner D Possinger K Ernst G Ruhstaller T. Comparison of orally administered cannabis extract and delta-9-tetrahydrocannabinol in treating patients with cancer-related anorexia-cachexia syndrome: a multicenter, phase III, randomized, double-blind, placebo-controlled clinical trial from the Cannabis-In-Cachexia-Study-Group. J Clin Oncol. (2006) 24:3394–400. 10.1200/JCO.2005.05.1847 16849753

[184] Brisbois TD de Kock IH Watanabe SM Mirhosseini M Lamoureux DC Chasen M. Delta-9-tetrahydrocannabinol may palliate altered chemosensory perception in cancer patients: results of a randomized, double-blind, placebo-controlled pilot trial. Ann Oncol. (2011) 22:2086–93. 10.1093/annonc/mdq727 21343383

[185] Bar-Sela G Zalman D Semenysty V Ballan E. The effects of dosage-controlled cannabis capsules on cancer-related cachexia and anorexia syndrome in advanced cancer patients: pilot study. Integr Cancer Ther. (2019) 18:1534735419881498. 10.1177/1534735419881498 31595793 PMC6785913

[186] Turcott JG Del Rocío Guillen Núñez M Flores-Estrada D Oñate-Ocaña LF Zatarain-Barrón ZL Barrón F. The effect of nabilone on appetite, nutritional status, and quality of life in lung cancer patients: a randomized, double-blind clinical trial. Support Care Cancer. (2018) 26:3029–38. 10.1007/s00520-018-4154-9 29550881

[187] Lindholm E Daneryd P Körner U Hyltander A Fouladiun M Lundholm K. Effects of recombinant erythropoietin in palliative treatment of unselected cancer patients. Clin Cancer Res. (2004) 10:6855–64. 10.1158/1078-0432.CCR-04-0373 15501962

[188] Bayliss TJ Smith JT Schuster M Dragnev KH Rigas JR. A humanized anti-IL-6 antibody. (ALD518) in non-small cell lung cancer. Expert Opin Biol Ther. (2011) 11:1663–8. 10.1517/14712598.2011.627850 21995322

[189] Tai Y-T Fulciniti M Hideshima T Song W Leiba M Li X-F. Targeting MEK induces myeloma-cell cytotoxicity and inhibits osteoclastogenesis. Blood. (2007) 110:1656–63. 10.1182/blood-2007-03-081240 17510321 PMC1975848

[190] Harrison C Kiladjian J-J Al-Ali HK Gisslinger H Waltzman R Stalbovskaya V. JAK inhibition with ruxolitinib versus best available therapy for myelofibrosis. N Engl J Med. (2012) 366:787–98. 10.1056/NEJMoa1110556 22375970

[191] Gramignano G Lusso MR Madeddu C Massa E Serpe R Deiana L. Efficacy of l-carnitine administration on fatigue, nutritional status, oxidative stress, and related quality of life in 12 advanced cancer patients undergoing anticancer therapy. Nutrition. (2006) 22:136–45. 10.1016/j.nut.2005.06.003 16459226

[192] Madeddu C Dessì M Panzone F Serpe R Antoni G Cau MC. Randomized phase III clinical trial of a combined treatment with carnitine + celecoxib ± megestrol acetate for patients with cancer-related anorexia/cachexia syndrome. Clin Nutr. (2012) 31:176–82. 10.1016/j.clnu.2011.10.005 22047681

[193] Pedersen BK Febbraio MA. Muscle as an endocrine organ: focus on muscle-derived interleukin-6. Physiol Rev. (2008) 88:1379–406. 10.1152/physrev.90100.2007 18923185

[194] Schnyder S Handschin C. Skeletal muscle as an endocrine organ: PGC-1α, myokines and exercise. Bone. (2015) 80:115–25. 10.1016/j.bone.2015.02.008 26453501 PMC4657151

[195] Manole E Ceafalan LC Popescu BO Dumitru C Bastian AE. Myokines as possible therapeutic targets in cancer cachexia. J Immunol Res. (2018) 2018:8260742. 10.1155/2018/8260742 30426026 PMC6217752

[196] Lach-Trifilieff E Minetti GC Sheppard K Ibebunjo C Feige JN Hartmann S. An antibody blocking activin type II receptors induces strong skeletal muscle hypertrophy and protects from atrophy. Mol Cell Biol. (2014) 34:606–18. 10.1128/MCB.01307-13 24298022 PMC3911487

[197] Mantovani G Macciò A Lai P Massa E Ghiani M Santona MC. Cytokine involvement in cancer anorexia/cachexia: role of megestrol acetate and medroxyprogesterone acetate on cytokine downregulation and improvement of clinical symptoms. Crit Rev Oncog. (1998) 9:99–106. 10.1615/CritRevOncog.v9.i2.10 9973244

[198] Yeh SS Wu SY Levine DM Parker TS Olson JS Stevens MR. The correlation of cytokine levels with body weight after megestrol acetate treatment in geriatric patients. J Gerontol A Biol Sci Med Sci. (2001) 56:M48–54. 10.1093/gerona/56.1.M48 11193233

[199] Nanjappa S Thai C Shah S Snyder M. Pharmacy report: megestrol acetate-induced adrenal insufficiency. Cancer Control. (2016) 23:167–9. 10.1177/107327481602300212 27218795

[200] Wen H-S Li X Cao Y-Z Zhang C-C Yang F Shi Y-M. Clinical studies on the treatment of cancer cachexia with megestrol acetate plus thalidomide. Chemotherapy. (2012) 58:461–7. 10.1159/000346446 23406994

[201] Khatib MN Gaidhane A Gaidhane S Quazi ZS. Ghrelin as a promising therapeutic option for cancer cachexia. Cell Physiol Biochem. (2018) 48:2172–88. 10.1159/000492559 30110683

[202] Al Massadi O López M Tschöp M Diéguez C Nogueiras R. Current Understanding of the hypothalamic ghrelin pathways inducing appetite and adiposity. Trends Neurosci. (2017) 40:167–80. 10.1016/j.tins.2016.12.003 28108113

[203] Ali S Chen J-A Garcia JM. Clinical development of ghrelin axis-derived molecules for cancer cachexia treatment. Curr Opin Support Palliat Care. (2013) 7:368–75. 10.1097/SPC.0000000000000012 24145681 PMC4121852

[204] Pietra C Takeda Y Tazawa-Ogata N Minami M Yuanfeng X Duus EM. Anamorelin HCl (ONO-7643), a novel ghrelin receptor agonist, for the treatment of cancer anorexia-cachexia syndrome: preclinical profile. J Cachexia Sarcopenia Muscle. (2014) 5:329–37. 10.1007/s13539-014-0159-5 25267366 PMC4248409

[205] Garcia JM Polvino WJ. Effect on body weight and safety of RC-1291, a novel, orally available ghrelin mimetic and growth hormone secretagogue: results of a phase I, randomized, placebo-controlled, multiple-dose study in healthy volunteers. Oncologist. (2007) 12:594–600. 10.1634/theoncologist.12-5-594 17522248

[206] Currow DC Skipworth RJ. The emerging role of anamorelin hydrochloride in the management of patients with cancer anorexia-cachexia. Fut Oncol. (2017) 13:1767–83. 10.2217/fon-2017-0141 28621564

[207] Ligresti A De Petrocellis L Di Marzo V. From phytocannabinoids to cannabinoid receptors and endocannabinoids: pleiotropic physiological and pathological roles through complex pharmacology. Physiol Rev. (2016) 96:1593–659. 10.1152/physrev.00002.2016 27630175

[208] Penna F Busquets S Toledo M Pin F Massa D López-Soriano FJ. Erythropoietin administration partially prevents adipose tissue loss in experimental cancer cachexia models. J Lipid Res. (2013) 54:3045–51. 10.1194/jlr.M038406 23966665 PMC3793608

[209] Kanzaki M Soda K Gin PT Kai T Konishi F Kawakami M. Erythropoietin attenuates cachectic events and decreases production of interleukin-6, a cachexia-inducing cytokine. Cytokine. (2005) 32:234–9. 10.1016/j.cyto.2005.10.002 16338141

[210] Fukawa T Yan-Jiang BC Min-Wen JC Jun-Hao ET Huang D Qian C-N. Excessive fatty acid oxidation induces muscle atrophy in cancer cachexia. Nat Med. (2016) 22:666–71. 10.1038/nm.4093 27135739

[211] Auger C Knuth CM Abdullahi A Samadi O Parousis A Jeschke MG. Metformin prevents the pathological browning of subcutaneous white adipose tissue. Mol Metab. (2019) 29:12–23. 10.1016/j.molmet.2019.08.011 31668383 PMC6728757

[212] Tseng Y-C Kulp SK Lai I-L Hsu E-C He WA Frankhouser DE. Preclinical investigation of the novel histone deacetylase inhibitor AR-42 in the treatment of cancer-induced cachexia. J Natl Cancer Inst. (2015) 107:djv274. 10.1093/jnci/djv274 26464423 PMC6280990

[213] Flint TR Janowitz T Connell CM Roberts EW Denton AE Coll AP. Tumor-induced IL-6 reprograms host metabolism to suppress anti-tumor immunity. Cell Metab. (2016) 24:672–84. 10.1016/j.cmet.2016.10.010 27829137 PMC5106372

[214] Beharry AW Sandesara PB Roberts BM Ferreira LF Senf SM Judge AR. HDAC1 activates FoxO and is both sufficient and required for skeletal muscle atrophy. J Cell Sci. (2014) 127:1441–53. 10.1242/jcs.136390 24463822 PMC3970557

[215] Gao W Xiong Y Li Q Yang H. Inhibition of toll-like receptor signaling as a promising therapy for inflammatory diseases: a journey from molecular to nano therapeutics. Front Physiol. (2017) 8:508. 10.3389/fphys.2017.00508 28769820 PMC5516312

[216] Chansrichavala P Chantharaksri U Sritara P Ngaosuwankul N Chaiyaroj SC. Atorvastatin affects TLR4 clustering via lipid raft modulation. Int Immunopharmacol. (2010) 10:892–9. 10.1016/j.intimp.2010.04.027 20472098

[217] Adams S Kozhaya L Martiniuk F Meng T-C Chiriboga L Liebes L. Topical TLR7 agonist imiquimod can induce immune-mediated rejection of skin metastases in patients with breast cancer. Clin Cancer Res. (2012) 18:6748–57. 10.1158/1078-0432.CCR-12-1149 22767669 PMC3580198

[218] Zahm CD Colluru VT McIlwain SJ Ong IM McNeel DG. TLR Stimulation during T-cell activation lowers PD-1 expression on CD8 T cells. Cancer Immunol Res. (2018) 6:1364–74. 10.1158/2326-6066.CIR-18-0243 30201735 PMC6215515

